# The genic view of hybridization in the Anthropocene

**DOI:** 10.1111/eva.13223

**Published:** 2021-03-26

**Authors:** Jente Ottenburghs

**Affiliations:** ^1^ Wildlife Ecology and Conservation Wageningen University & Research Wageningen The Netherlands; ^2^ Forest Ecology and Forest Management Wageningen University & Research Wageningen The Netherlands

**Keywords:** climate change, conservation, genomics, hybrid zones, introgression, speciation

## Abstract

Human impact is noticeable around the globe, indicating that a new era might have begun: the Anthropocene. Continuing human activities, including land‐use changes, introduction of non‐native species and rapid climate change, are altering the distributions of countless species, often giving rise to human‐mediated hybridization events. While the interbreeding of different populations or species can have detrimental effects, such as genetic extinction, it can be beneficial in terms of adaptive introgression or an increase in genetic diversity. In this paper, I first review the different mechanisms and outcomes of anthropogenic hybridization based on literature from the last five years (2016–2020). The most common mechanisms leading to the interbreeding of previously isolated taxa include habitat change (51% of the studies) and introduction of non‐native species (34% intentional and 19% unintentional). These human‐induced hybridization events most often result in introgression (80%). The high incidence of genetic exchange between the hybridizing taxa indicates that the application of a genic view of speciation (and introgression) can provide crucial insights on how to address hybridization events in the Anthropocene. This perspective considers the genome as a dynamic collection of genetic loci with distinct evolutionary histories, giving rise to a heterogenous genomic landscape in terms of genetic differentiation and introgression. First, understanding this genomic landscape can lead to a better selection of diagnostic genetic markers to characterize hybrid populations. Second, describing how introgression patterns vary across the genome can help to predict the likelihood of negative processes, such as demographic and genetic swamping, as well as positive outcomes, such as adaptive introgression. It is especially important to not only quantify how much genetic material introgressed, but also what has been exchanged. Third, comparing introgression patterns in pre‐Anthropocene hybridization events with current human‐induced cases might provide novel insights into the likelihood of genetic swamping or species collapse during an anthropogenic hybridization event. However, this comparative approach remains to be tested before it can be applied in practice. Finally, the genic view of introgression can be combined with conservation genomic studies to determine the legal status of hybrids and take appropriate measures to manage anthropogenic hybridization events. The interplay between evolutionary and conservation genomics will result in the constant exchange of ideas between these fields which will not only improve our knowledge on the origin of species, but also how to conserve and protect them.

## 1 INTRODUCTION

Human activities are affecting global environmental processes and patterns, from rapid climate change and radical land‐use changes to the introduction of exotic species, prompting some scientists to declare that we have entered a new era: the Anthropocene. The exact start of the Anthropocene has been a matter of debate and its onset has been connected to different events, such as the rise of deforestation and agriculture (Ruddiman, 2003), the Columbian Exchange of species between the Old World and the New World (Lewis & Maslin, 2015), the Industrial Revolution in the 1800s (Crutzen, 2002), and the population growth and industrialization during the mid‐20th century (Steffen et al., 2015). Based on the increase in the use of certain materials (e.g., aluminum, plastics, concrete), the nuclear fallout of atomic bomb testing, the geochemical signatures of particular compounds (e.g., polyaromatic hydrocarbons and pesticides), and the atmospheric rise in carbon concentrations, Waters et al., (2016) proposed a lower boundary for the Anthropocene during the mid‐20th century. In this review, I will follow this definition to provide a clear distinction between hybridization events that occurred before or during the Anthropocene.

Anthropogenic developments affect the distribution of numerous species, often resulting in secondary contact between previously allopatric taxa. If these taxa are closely related and reproductive isolation is incomplete, hybridization might occur. In this review, I will define hybridization following Arnold (1997), namely the situation in which “two populations of individuals that are distinguishable on the basis of one or more heritable characters overlap spatially and temporally and cross to form viable and at least partially fertile offspring.” These hybrid interactions can have detrimental consequences for the interbreeding populations, such as genetic swamping or extinction by hybridization (Rhymer & Simberloff, 1996; Todesco et al., 2016). This is why conservationists have established guidelines to deal with such cases of human‐induced hybridization (Allendorf et al., 2001). However, hybridization can also provide ecological and evolutionary opportunities, such as the origin of new hybrid species (Mallet, 2007; Ottenburghs, 2018) or the exchange of adaptive genetic variation (Arnold & Kunte, 2017; Hedrick, 2013). It is thus important to determine the balance between these potential detrimental and beneficial consequences when devising effective conservation strategies.

In the following sections, I first review the literature on anthropogenic hybridization from the last five years (2016–2020) to identify the most common mechanisms and outcomes of human‐mediated hybridization events (see Table 1, extending the search strategy from Grabenstein & Taylor, 2018). Next, I introduce the “genic view of speciation,” a concept that has shaped recent research agendas in speciation genomics (Campbell et al., 2018; Ravinet et al., 2017; Wu, 2001). This viewpoint focuses on the heterogenous nature of genetic differentiation and introgression across the genome. The insight that different genomic regions tell different evolutionary stories needs to be taken into account when dealing with anthropogenic hybridization events. Specifically, it has important consequences for (1) the development of molecular markers, (2) the quantification of (adaptive) introgression patterns, and (3) the legal status of hybrids.

**TABLE 1:** Overview of studies on anthropogenic hybridization events published in the last five years (2016–2020)

Species	Location	Mechanism	Outcome	Molecular markers	Reference
Plants					
*Centaurea seridis* *Centaurea aspera*	Spain	Habitat Change	F1 hybrids (triploids)	NA	Garmendia et al., (2018)
*Eucalyptus tetrapleura*	Australia	Habitat Change	Introgression	RADseq	Rutherford et al., (2018)
*Odontarrhena* spp.	Albania	Habitat Change	Introgression	AFLP‐fingerprinting	Coppi et al., (2020)
*Phragmites australis* *Phragmites mauritanius*	Southern Africa	Habitat Change	Introgression	Chloroplast DNA Microsatellites	Canavan et al., (2018)
	Nevada, USA	Introduction	F1 hybrids		Saltonstall et al., (2016)
*Quercus durata* *Quercus berberidifolia*	California, USA	Habitat Change (fire frequency)	Introgression	Microsatellites	Ortego et al., (2017)
*Rhododendron ferrugineum* *Rhododendron hirsutum*	Italy	Habitat Change	Introgression	Chloroplast DNA Microsatellites	Bruni et al., (2016)
*Taraxacum calanthodium* *Taraxacum lugubre*	China	Pollination by introduced bees	Introgression	Microsatellites	Peng et al., (2018)
Insects					
*Bactrocera tryoni* *Bactrocera aquilonis*	Australia	Habitat Change (horticulture)	Introgression	RADseq	Popa‐Báez et al., (2020)
*Helicoverpa armigera* *Helicoverpa zea*	Brazil	Introduction (invasive species)	Introgression * (adaptive)	Whole Genome	Valencia‐Montoya et al., (2020)
*Phaulacridium marginale* *Phaulacridium otagoense*	New Zealand	Habitat Change (deforestation)	Introgression	Mitochondrial and nuclear loci	Sivyer et al., (2018)
Amphibians					
*Bufo woodhousii* *Bufo microscaphus*	Southwest USA	Habitat Change	Introgression	Microsatellites	Wooten et al., (2019)
*Hyperolius thomensis* *Hyperolius molleri*	Sao Tomé Island	Habitat Change (deforestation)	Introgression	mtDNA RADseq	Bell and Irian (2019)
*Lissotriton vulgaris meridionalis* *Lissotriton vulgaris vulgaris*	Italy	Introduction	F1 hybrids	Mitochondrial and nuclear loci	Dubey et al., (2019)
*Pelophylax* spp.	Switzerland	Introduction	F1 hybrids	mtDNA Microsatellites	Dufresnes et al., (2018)
*Rana pipiens*	Southern USA	Introduction	Introgression	mtDNA Microsatellites	O’Donnell et al., (2017)
*Triturus cristatus* *Triturus carnifex*	The Netherlands	Introduction	Introgression	Mitochondrial and nuclear loci	Wielstra et al., (2016)
Reptiles					
*Pelodiscus* spp.	China	Introduction (farmed turtles)	Introgression	Mitochondrial and nuclear loci	Gong et al., (2018)
*Sternotherus depressus* *Sternotherus peltifer*	Alabama, USA	Habitat Change	Introgression (unidirectional)	RADseq	Scott et al., (2019)
Fish					
*Archosargus probatocephalus* *Archosargus rhomboidalis*	Florida, USA	Habitat Change	F1 hybrids	mtDNA & nDNA microsatellites	Seyoum et al., (2020)
*Alosa alosa* *Alosa fallax*	France	Habitat Change	Introgression	Mitochondrial and nuclear loci	Taillebois et al., (2020)
*Alosa pseudoharengu*	Connecticut, USA	Introduction	Introgression	RADseq	Reid et al., (2020)
*Catostomus discobolus* *Catostomus ardens*	USA	Habitat Change	F1 hybrids	Mitochondrial and nuclear loci	Bangs et al., (2017)
*Cobitis magnostriata* *Cobitis minamorii oumiensis*	Japan	Habitat Change	Reproductive Interference	mtDNA	Morii et al., (2018)
*Colpichthys regis* *Colpichthys hubbsi*	California, USA	Habitat Change	Introgression (unidirectional)	mtDNA Microsatellites	Lau and Jacobs (2017)
*Coregonus lavaretus* (wild and stocked)	Finland	Introduction & Habitat Change	Introgression	Microsatellites	Huuskonen et al., (2017)
*Coregonus* spp.	Switzerland	Habitat Change (eutrophication)	Introgression	RADseq	Feulner and Seehausen (2019)
*Cottus* sp. *Cottus cognatus*	Canada	Habitat Change	Introgression	mtDNA Microsatellites	Rudolfsen et al., (2019)
*Etheostoma osburni* *Etheostoma variatum*	West Virginia, USA	Habitat Change	Introgression	Microsatellites	Gibson et al., (2019)
*Fundulus grandis* *Fundulus heteroclitus*	Mexico	Habitat Change	Introgression * (adaptive)	Whole Genome	Oziolor et al., (2019)
*Gila cypha* *Gila robusta*	Colorado, USA	Habitat Change	Introgression	RADseq	Chafin et al., (2019)
*Macquaria ambigua* (wild and stocked)	Australia	Introduction	Introgression	RADseq	Beheregaray et al., (2017)
*Micropterus* spp.	Southern USA	Introduction	Introgression	Mitochondrial and nuclear loci	Bangs et al., (2018)
*Oncorhynchus chrysogaster* *Oncorhynchus mykiss*	Mexico	Introduction (aquaculture)	Introgression	RADseq	Escalante et al., (2020)
*Oncorhynchus clarkii* *Oncorhynchus mykiss*	USA	Introduction	Introgression	Allozymes, SNPs and microsatellites	Muhlfeld et al., (2017)
*Oncorhynchus tshawytscha*	Washington, USA	Habitat Change	F1 hybrids	SNP markers	Fraser et al., (2020)
*Oreochromis niloticus*	Ethiopia	Introduction	Introgression	Microsatellites	Tibihika et al., (2020)
*Parachondrostoma toxostoma* *Chondrostoma nasus*	France	Habitat Change	Introgression	mtDNA Microsatellites	Guivier et al., (2019)
*Salvelinus alpinus* *Salvelinus fontinalis*	Sweden	Introduction	Introgression	mtDNA Microsatellites	Faulks and Östman (2016)
*Salvelinus fontinalis* (wild and stocked)	New York, USA	Introduction	Introgression	Microsatellites	Bruce et al., (2020)
*Salvelinus fontinalis* *Salvelinus confluentus*	Oregon, USA	Introduction & Habitat Change	F1 hybrids (morphology)	NA	Howell (2018)
*Sander vitreus* *Sander Canadensis*	Canada	Introduction & Habitat Change	Introgression	RADseq	Graham et al., (2020)
*Tropheus moorii* (two lineages)	Lake Tanganyika	Habitat Change (lake levels)	Introgression	mtDNA, AFLP, and Microsatellites	Sefc et al., (2017)
Birds					
*Alectoris rufa* *Alectoris chukar*	Italy	Introduction (hunting)	Introgression	mtDNA	Forcina et al., (2020)
*Francolinus francolinus* (subspecies)	Pakistan	Introduction	Introgression	mtDNA Microsatellites	Forcina et al., (2018)
*Mycteria cinerea* *Mycteria leucocephala*	Singapore	Introduction (captive hybrids)	Introgression	RADseq	Baveja et al., (2019)
*Vermivora chrystoptera* *Vermivora cyanoptera*	Canada	Habitat Change	F1 Hybrids	mtDNA	Moulton et al., (2017)
Mammals					
*Aepyceros melampus petersi* *Aepyceros melampus melampus*	Namibia & South Africa	Introduction	Introgression	Microsatellites	Miller et al., (2020)
*Callithrix* spp.	Brazil	Introduction	Introgression	NA	Malukiewicz (2019)
*Canis lupus* *Canis lupus familiaris*	Italy	Introduction (domestic dogs)	Introgression	Nuclear loci	Salvatori et al., (2019)
*Cervus elaphus* *Cervus nippon*	Scotland	Introduction	Introgression	SNP genotyping	McFarlane et al., (2020)
*Cervus elaphus*	Spain & Portugal	Introduction (non‐native deer)	Introgression	mtDNA Microsatellites	Queirós et al., (2020)
*Damaliscus pygargus pygargus* *Damaliscus pygargus phillipsi*	South Africa	Introduction	F1 hybrids	Microsatellites	Van Wyk et al., (2017)
*Felis silvestris* *Felis catus*	Europe	Introduction (domestic cats)	Introgression	SNP genotyping Microsatellites	Tiesmeyer et al., (2020)
*Gazella bennettii* *Gazella subgutturosa*	Iran	Introduction	F1 Hybrids	Mitochondrial and nuclear loci	Fadakar et al., (2020)
*Sus scrofa* (wild and domesticated)	Japan	Introduction (escaped animals)	Introgression	mtDNA	Anderson et al., (2019)
Other taxa					
*Daphnia pulex* (different lineages)	Canada	Habitat Change	Introgression	mtDNA Microsatellites	Millette et al., (2020)
*Daphnia longispina* *Daphnia galeata*	Switzerland	Habitat Change (eutrophication)	Introgression	mtDNA Microsatellites	Alric et al., (2016)
*Pomacea canaliculata* *Pomacea maculata*	Brazil & Uruguay	Introduction	Introgression	Nuclear markers	Glasheen et al., (2020)

Note

These studies were found in a systematic literature search of Google Scholar and the Web of Science, using the combined search terms: ‘anthropogenic AND disturb* AND hybrid*’OR ‘habitat AND chang* AND hybrid*’ OR ‘human AND chang* AND hybrid*’ OR ‘environment* AND chang* AND hybrid*’ NOT climate NOT introduc* NOT zone* (following Grabenstein & Taylor).

## 2 MECHANISMS OF ANTHROPOGENIC HYBRIDIZATION

**FIGURE 1:**
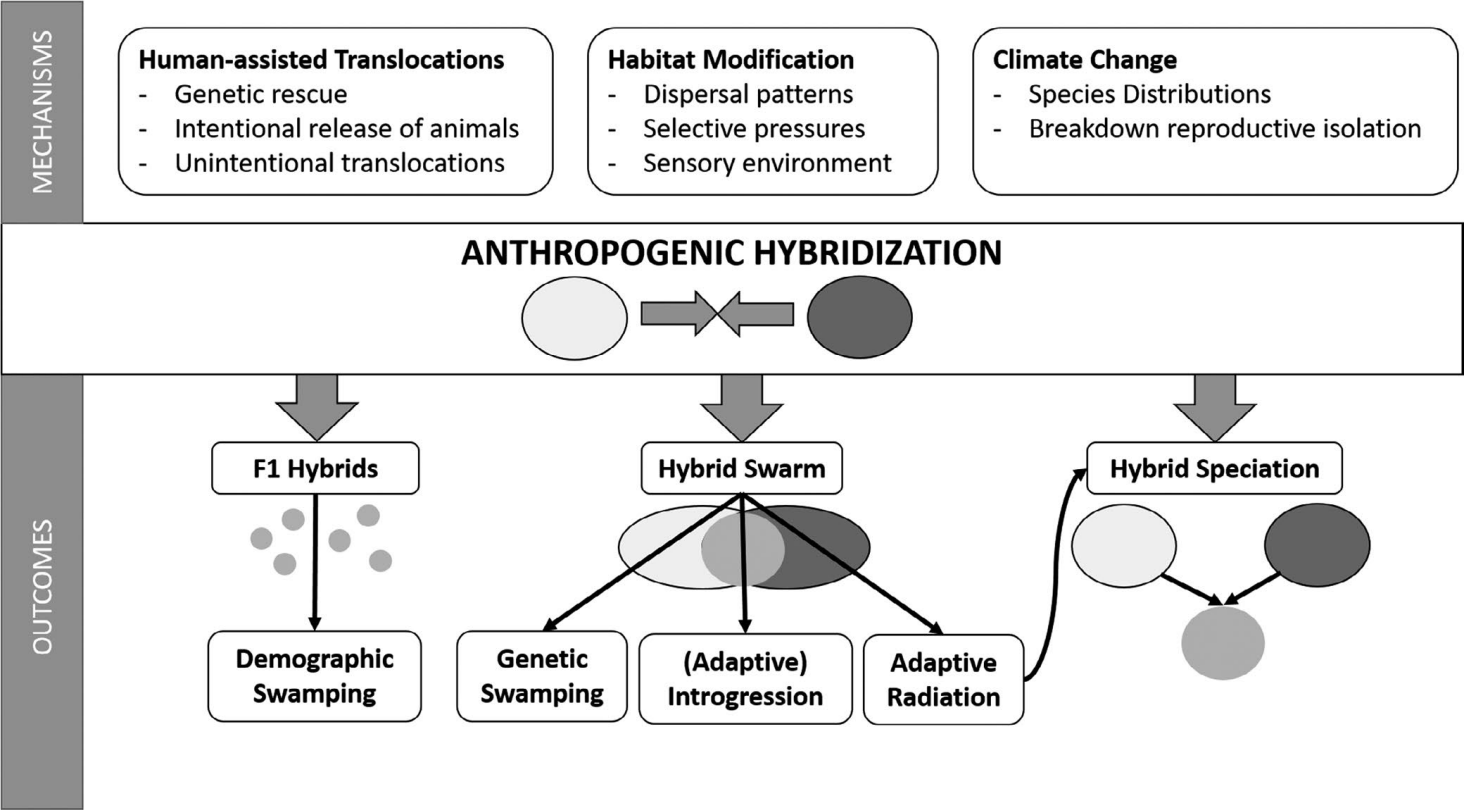
Mechanisms and outcomes of anthropogenic hybridization. Mechanisms that can result in hybridization include human‐assisted translocations (both intentional and unintentional), habitat modification and climate change. Anthropogenic hybridization events can result in the formation of first‐generation (F1) hybrids, a hybrid swarm or a hybrid species. If the production of F1 hybrids interferes with the reproductive output of the parental species, demographic swamping can occur. A hybrid swarm can lead to (adaptive) introgression, genetic swamping or provide the raw material for an adaptive radiation (potentially including the origin of a hybrid species)

**FIGURE 2:**
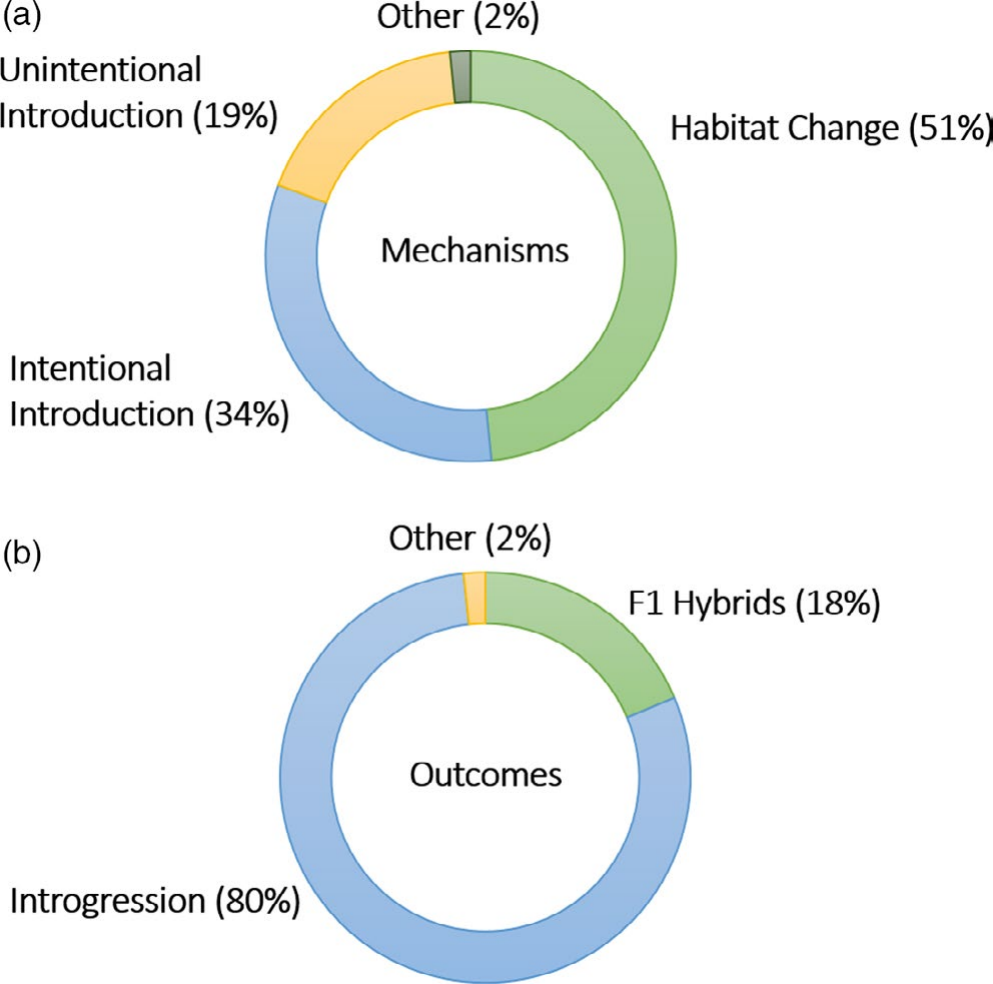
The most common mechanisms and outcomes of anthropogenic hybridization, based on a literature review of the last five years (2016–2020). Habitat change is the most prevalent mechanism behind human‐induced hybridization events, followed by intentional and unintentional translocations of organisms. The vast majority of anthropogenic hybridization events results in introgression

There are three main mechanisms of anthropogenic hybridization (Figure 1), namely human‐assisted translocations, habitat modifications, and climate change (Crispo et al., 2011; Grabenstein & Taylor, 2018). Translocations or introductions of certain taxa can be intentional, such as in genetic rescue programs. This strategy is used to restore genetic diversity and reduce the extinction risk of small, isolated, and often inbred populations. About 90 percent of these genetic rescue attempts have been successful, indicating that human‐mediated hybridization can be an important tool in conservation (Frankham, 2015). In this review, however, I focus on species translocations and introductions that are unrelated to conservation efforts. My literature search uncovered 31 papers (out of 59 studies, 53%) that involved the introduction of non‐native species (Figure 2a). Some of these cases involved intentional movement of organisms, such as the release of game‐farm birds for hunting purposes (Forcina et al., 2020), the stocking of fish populations with captive‐bred animals (Beheregaray et al., 2017; Bruce et al., 2020), or the translocation of large mammals between African game reserves (Grobler et al., 2018; Miller et al., 2020; van Wyk et al., 2019). Other human‐assisted translocations were unintentional, such as the transport of aquatic organisms in ship hulls (Oziolor et al., 2019) or plant seeds by cargo or passenger transport, sometimes resulting in cryptic invasions and hybridization events (Morais & Reichard, 2018). In addition, domestic animals that escape from captivity often interbreed with their wild relatives (Anderson et al., 2019; Salvatori et al., 2019; Tiesmeyer et al., 2020). Species translocations or introductions—both intentional and unintentional—leading to hybridization events have thus been documented in a variety of taxa and are expected to increase as humans continue to move species across the globe (Crispo et al., 2011).

Anthropogenic hybridization can also be the outcome of habitat modifications (Grabenstein & Taylor, 2018). Edgar Anderson (1948) coined the phrase “hybridization of the habitat” to indicate that human disturbances of the environment can lead to the production of hybrid offspring. Indeed, 30 studies (51%) attributed hybridization events to human‐induced habitat changes (Figure 2a). In several plant species, the rate of hybridization increased with the level of disturbance in the area, such as the concentration of heavy metals (Coppi et al., 2020) or the frequency of wildfires (Ortego et al., 2017). Hybridization can also be the outcome of homogenization of the habitat or the construction of landscape features that facilitate dispersal (e.g., roads and verges), culminating in contact between previously isolated populations (Bangs et al., 2017; Carantón‐Ayala et al., 2018; van Hengstum et al., 2012). For instance, deforestation has led to hybridization between certain insect (Sivyer et al., 2018) and amphibian populations (Bell & Irian, 2019). Moreover, habitat disturbances can affect selective pressures, allowing hybrids to thrive in new environments that are not accessible for the parental populations (Arnold et al., 2012; Arnold & Martin, 2010). For example, hybrids between cave salamanders (*Hydromantes ambrosii* and *Hydromantes italicus*) with transgressive phenotypes could expand into more harsh environments with higher food availability (Ficetola et al., 2019). Human‐mediated actions can also influence the sensory environment in a number of ways, such as pollutants that alter chemical signaling (Smadja & Butlin, 2009) or anthropogenic noise that interferes with auditory communication (Slabbekoorn et al., 2010; Slabbekoorn & Ripmeester, 2008). A well‐studied example concerns the effect of increased lake eutrophication on species recognition in fish (Alexander et al., 2017; Vonlanthen et al., 2012). Eutrophication decreases water clarity, leading to a breakdown of prezygotic barriers because individuals cannot discriminate between conspecifics and heterospecifics in the turbid waters (Seehausen et al., 1997). As human‐mediated habitat modifications continue to change the environmental and sensory conditions for numerous taxa, more hybridization events are expected to arise in the near future (Crispo et al., 2011; Grabenstein & Taylor, 2018).

Climate change can be seen as a special case of habitat change and can affect hybridization dynamics in a myriad of ways (Chunco, 2014). Both latitudinal and altitudinal range shifts are expected to occur, leading to secondary contact between previously isolated species (Larson et al., 2019; Parmesan, 2006; Taylor et al., 2015). Such distributional shifts and consequent hybridization events have already been documented, for instance between polar bears (*Ursus maritimus*) and grizzly bears (*U. arctos*) in the Arctic (Kelly et al., 2010) and between species of *Glaucomys* flying squirrels (Garroway et al., 2010), and will be observed more often in the future (Chunco, 2014; Taylor et al., 2015). Apart from distributional changes, climate change might also result in the breakdown of reproductive isolation mechanisms between sympatric species, such as the disappearance of temporal isolation due to phenological changes (Vallejo‐Marín & Hiscock, 2016). Climate change will thus alter population dynamics in time and space, culminating in more hybridization events.

## 3 OUTCOMES OF ANTHROPOGENIC HYBRIDIZATION

Regardless of the underlying mechanism, anthropogenic hybridization can have several outcomes, which are mainly determined by the level of genetic divergence and the nature of reproductive isolation between the interacting species (Figure 1). In some cases, the hybridization event is limited to first‐generation (F1) hybrids (11 studies, 19%, Figure 2b), such as the production of triploid hybrids between diploid and tetraploid *Centaurea* species in Spain (Garmendia et al., 2018) or the occasional hybrid between subspecies of the smooth newt (*Lissotriton vulgaris*) in Italy (Dubey et al., 2019). The absence of second‐generation hybrids or backcrosses might be due to the lower fitness of F1 hybrids (e.g., decreased fertility) or the lower sensitivity of certain molecular markers that fail to identify later‐generation hybrids. Another possible outcome of human‐induced hybridization is a hybrid swarm: a population of fertile hybrids that survived past the first hybrid generation, followed by interbreeding between hybrid individuals and backcrossing with their parental populations. Hybrid swarms generally form within a hybrid zone, an area where two populations overlap spatially and temporally and produce viable and at least partially fertile offspring within a restricted area (e.g., Carney et al., 2000; Fisher et al., 2006). In the majority of studies (47 studies, 80%), the production of hybrids resulted in the exchange of genetic material through backcrossing with parental species (i.e., introgression, Figure 2b). On a longer timeframe, the fate of these hybrid swarms is difficult to predict. The mixing of different genetic lineages might lead to the extinction of particular lineages through species collapse (Zhang et al., 2019) or it might provide the raw material for an adaptive radiation (Marques et al., 2019; Seehausen, 2004). In theory, hybrid species can emerge from hybrid swarms, but this speciation process is typically too slow to observe on a short timescale (Lamichhaney et al., 2018; Ottenburghs, 2018; Seehausen, 2004). There are, however, some exceptions, such as the Oxford ragwort (*Senecio squalidus*) that originated from the interbreeding of two Italian species (*S. aethnensis* and *S. chrysanthemifolius*) in British gardens at the turn of the 18^th^ century (Nevado et al., 2020).

On a population level, different combinations of hybrid fitness and hybridization rate can lead to drastically different outcomes in an anthropogenic hybridization event. First, if hybrid fitness is comparable to the fitness of parents (i.e., outbreeding depression is low) and hybrids show higher population growth rates than the parental taxa, genetic swamping might occur in which the parental taxa are replaced by hybrids (Rhymer & Simberloff, 1996). This might be occurring in West Virginia (USA) where hybrids between candy darters (*Etheostoma osburni*) and variegate darters (*E. variatum*) are replacing the now endangered candy darter (Gibson et al., 2019). A special case of potential genetic swamping concerns the release of actively managed species, such as fish stocks and game birds (Randi, 2008). These artificially selected organisms often differ genetically from their wild conspecifics which can result in outbreeding depression when they interbreed (Muhlfeld et al., 2009). Moreover, the strong selection pressures in captivity might lead to low genetic diversity and inbreeding depression (Willoughby et al., 2015). Hybridization with these maladapted individuals can lower the average fitness of wild populations. Although most studies documented introgression and warn for the possibility of genetic swamping, few studies directly quantified the likelihood of genetic swamping in their study system. This important knowledge gap should be addressed with more detailed analyses, complemented with modeling studies, to determine crucial tipping points in introgression levels that can lead to genetic swamping.

If hybrid fitness is strongly reduced compared to the parental taxa (i.e., outbreeding depression is high) and hybridization rates are high, parental taxa will waste reproductive effort when hybridizing. This situation can lead to demographic swamping where parental taxa decline due to decreased population growth rates (Wolf et al., 2001). This situation has been described for the Australian native variable groundsel (*Senecio pinnatifolius*) and the invasive Madagascar ragwort (*S. madagascariensis*). Hybridization rates between these species are very high, but hybrids are not viable. As the invasive plant increases in abundance, the native plant continues to waste reproductive effort and will start declining in numbers (Prentis et al., 2007). Another example concerns reproductive interference between two spined loaches (*Cobitis magnostriata* and *C. minamorii oumiensis*) that could lead to the decline of the latter species (Morii et al., 2018). Between the extremes of genetic and demographic swamping, there is a continuum of introgression patterns that are neutral or benefit one or both hybridizing taxa.

## 4 THE GENIC VIEW OF SPECIATION AND INTROGRESSION

This overview of the different mechanisms and outcomes of anthropogenic hybridization highlights the difficulty in predicting the future developments of hybrid interactions and devising appropriate conservation measures. Recent developments in genomic tools have led to more accurate detection of hybrids and the quantification of introgression patterns (McFarlane & Pemberton, 2019), but an overarching framework is needed to interpret these findings and translate them to successful conservation strategies. Here, speciation genomics and the study of pre‐Anthropocene hybridization events can provide additional insights (Campbell et al., 2018; Taylor et al., 2015). In particular, the genic view of speciation has drastically changed the way evolutionary biologists study the process of speciation (Bazykin, 1969; Key, 1968; Wu, 2001) and this perspective can be applied to several conservation genomic questions.

Speciation research has long been dominated by the Biological Species Concept (BSC) which focuses on reproductive isolation between diverging lineages (Mayr, 1963). This concept assumed that species differentiation is controlled by a large number of genetic loci and that the whole genome functions as an integrated and cohesive genetic unit. Hybridization and consequent genetic exchange were thought to destroy this integrity and break up “co‐adapted gene complexes.” This perspective is still widely followed by conservationists that describe introgressive hybridization as “genetic erosion” (Chafin et al., 2019) or “genetic pollution” (Wielstra et al., 2016). However, genomic studies have overturned the idea of the genome as a cohesive genetic unit that can be destroyed by hybridization. Instead, the genome can better be regarded as a dynamic collection of genetic loci with separate, but entangled evolutionary histories. Moreover, reproductive isolation is often controlled by epistatic interactions between a few genetic loci (Ravinet et al., 2017; Wu, 2001).

This new perspective is nicely illustrated by recent studies that showed how genetic differentiation between diverging lineages is heterogeneously distributed across the genome, often concentrated in a few genomic regions, so‐called “islands of differentiation” (Wolf & Ellegren, 2017). The genomic islands may harbor loci that contribute to reproductive isolation, and these so‐called barrier loci are thus less likely to introgress compared to neutral loci. Consequently, barrier loci and closely linked genomic regions will diverge while introgression homogenizes the rest of the genome (Ravinet et al., 2017). An alternative explanation for the origin of genomic islands concerns linked selection, which comprises two processes: background selection and genetic hitchhiking (Burri, 2017). Background selection refers to purifying selection against recurring deleterious mutations, while genetic hitchhiking occurs when positive selection on a variant result in the selection for the genomic region in which this advantageous variant resides. As the advantageous variant increases in frequency, the loci linked to this variant hitchhike along (Sendell‐Price et al., 2020). Regardless of the underlying process—barrier loci or linked selection—the end result is a heterogenous genomic landscape of differentiation, which affects several aspects of conservation genomics, including (1) the development of molecular markers, (2) the study of introgression, and (3) the legal status of hybrids.

## 5 DEVELOPMENT OF MOLECULAR MARKERS

A first step in the assessment of an anthropogenic hybridization event involves determining the composition of the hybrid population in terms of parental individuals, first‐generation hybrids (F1), second‐generation hybrids (F2), backcrosses, etc. For example, a mixture of different generational hybrids indicates the formation of a hybrid swarm, whereas the absence of backcrosses and second‐generation hybrids suggests selection against hybrids. Most conservation genetic studies use a set of variable genetic markers (e.g., microsatellites) to quantify the genetic make‐up of different individuals using software packages such as *STRUCTURE* (Pritchard et al., 2000) or *ADMIXTURE* (Alexander et al., 2009). Next, this genetic make‐up can be compared with simulated data to test the power of discriminating between different levels of admixture (Anderson, 2008). The resulting composition of the hybrid population provides the basis for subsequent analyses and potential conservation measures.

The use of a few genetic markers works well to identify early‐generation hybrids and backcrosses, but can run into several issues (McFarlane & Pemberton, 2019). First, the determination of a threshold to discriminate between hybrid and parental species can be problematic. Population assignment algorithms calculate an admixture score (Q) for individuals where a score of 0 or 1 indicates a purebred individual from one of the parental populations. However, due to errors in genotyping or the presence of nondiagnostic markers, most individuals will have a score that is not exactly 0 or 1. But how does one discriminate between these errors and actual hybrids (which have a score between 0 and 1)? Generally, a threshold is used to delimit the parental classes, but this threshold varies between studies, ranging from 0.8 (Schulte et al., 2012) to 0.99 (Galaverni et al., 2017), and the choice of the threshold depends on the number of hybrid classes one recognizes. Using a threshold of 0.8 would, for instance, assign some later‐generation backcrosses to a parental class. A second issue of using few genetic makers for hybrid detection concerns homozygous loci. By chance, several generations of backcrossing can result in homozygous genetic markers for some individuals, which will consequently not be recognized as hybrids or backcrosses (Boecklen & Howard, 1997).

These two issues—setting a realistic admixture threshold and the presence of homozygous markers—can often be solved by adding more markers. Indeed, genomic studies can more confidently identify hybrids and later‐generation backcrosses, leading to more sound conclusions about the hybridization dynamics (Lemopoulos et al., 2019; McFarlane et al., 2020; McFarlane & Pemberton, 2019). For instance, analyses based on microsatellite markers suggested that hybridization between mallards (*Anas platyrhynchos*) and American black ducks (*Anas rubripes*) might lead to genetic extinction of the latter species (Mank et al., 2004). However, genomic studies of this system revealed little gene flow between the species, indicating that hybridization is not threatening the genetic integrity of the American black duck (Lavretsky et al., 2019, 2020). Similarly, McFarlane et al., (2020) investigated the sensitivity of microsatellites and RADseq (restriction site‐associated DNA sequencing) to discriminate between different hybrid classes of red deer (*Cervus elaphus*) and Japanese sika (*C. nippon*). The RADseq data were able to identify more advanced backcrosses compared to the microsatellites, leading to a more fine‐grained picture of introgression dynamics between these species.

The discrepancy between microsatellite markers and genomic data can partly be explained by the underlying genomic landscape of differentiation. The random selection of a few genetic markers might result in a marker set that only captures the undifferentiated section of the genome, missing the genomic islands of differentiation. Genomic sequencing methods, such as RADseq, cover a larger proportion of the genome compared to microsatellites and can be used to develop diagnostic markers. For instance, Taillebois et al., (2020) developed 77 species‐specific SNPs (single nucleotide polymorphisms) that could detect hybrids and backcrosses between allis shad (*Alosa alosa*) and twaite shad (*A. fallax*) up to the third generation. Similar marker sets and sequencing protocols have been developed for other study systems (Feulner & Seehausen, 2019; Vaux et al., 2021; Wielstra et al., 2016) and show that conservationists do not always need whole genome sequencing data to reconstruct the entire genomic landscape. This conclusion is further supported by evolutionary studies that used less powerful sequencing methods (e.g., RADseq or ultraconserved elements) to explore the genomic landscape of differentiation (Battey, 2019; Bourgeois et al., 2020; Oswald et al., 2019; Plomion et al., 2018). There are, however, certain situations where these methods do not provide the necessary resolution to investigate genetic differentiation across the genome, such as polyploids (Bourke et al., 2018; Clark et al., 2019), large genome sizes (Lowry et al., 2016), or populations with large‐scale demographic changes (Arnold et al., 2013). It is thus important to be conscious about the potential biases of reduced representation methods, such as RADseq (reviewed in Andrews et al., 2016). Nonetheless, being aware of the heterogenous nature of the underlying genomic landscape will already lead to a more conscious selection of diagnostic markers, even if not using whole genome sequencing.

## 6 PATTERNS OF ANTHROPOGENIC INTROGRESSION

Hybridization often results in introgression: the exchange of genetic material between populations through backcrossing (Ottenburghs, Kraus, et al., 2017; Ottenburghs, Megens, et al., 2017; Taylor & Larson, 2019). Introgression can have detrimental effects, such as loss of genetic integrity, speciation reversal (Seehausen et al., 2008), and the genetic extinction of certain taxa (Rhymer & Simberloff, 1996). However, introgression can be beneficial by facilitating the exchange of adaptive loci and increasing genetic diversity (Arnold & Kunte, 2017; Hedrick, 2013), possibly improving the adaptive potential of a population or species (Funk et al., 2019; Milot et al., 2020). Indeed, several authors have suggested that hybridization can be used as an effective conservation measure (Chan et al., 2019; vonHoldt et al., 2018). From a conservation point of view, we are thus faced with a difficult dilemma when assessing a human‐induced hybridization event: should we prevent potential genetic extinction with conservation measures (e.g., culling hybrids) or should we not intervene to provide the opportunity for adaptive introgression and an increase in genetic diversity?

The positive effects of introgressive hybridization have been well‐documented in several genetic rescue programs (Frankham, 2015; Whiteley et al., 2015), but does it also occur in unintentional anthropogenic hybridization events? A literature survey reported that the majority of human‐induced hybridization events led to an increased extinction risk of the parental species (Todesco et al., 2016), indicating that introgression was mainly detrimental. The discrepancy between the success of intentional genetic rescue programs and the detrimental effects of unintentional anthropogenic hybridization events can be partly explained by the genetic divergence between the hybridizing taxa (Whiteley et al., 2015). Genetic rescue involves the carefully planned supplementation of a small inbred population with individuals from other populations that belong to the same (Heber et al., 2013; Miller et al., 2012) or a closely related subspecies (Harrisson et al., 2016; Pimm et al., 2006) in order to restore genetic diversity. Because the genetic distance between the local and supplemented individuals is low, it is unlikely that the resulting hybrids will suffer from outbreeding depression (i.e., decreased fitness of hybrids relative to their parents). In fact, hybrids in genetic rescue programs often exhibit a temporary increase in fitness due to the masking of rare deleterious alleles (Pickup et al., 2013; Weeks et al., 2017). In contrast to genetic rescue interventions, anthropogenic hybridization events often occur between more distantly related taxa, increasing the likelihood of outbreeding depression due to negative epistatic interactions between divergent loci (Edmands, 1999). Indeed, the majority of studies in my literature search (43 out of 59 studies, 73%) involved different species.

Speciation genomics—particularly the study of pre‐Anthropocene hybrid zones—has shown that introgression varies across the genome (Payseur, 2010). Genetic loci can roughly be divided into three categories: (1) neutral loci that flow freely between taxa, (2) deleterious loci that contribute to reduced fitness in hybrids and inhibit introgression, and (3) beneficial loci that confer an adaptive advantage and increase in frequency following introgression. Admixed genomes can be seen as a mosaic of these three categories, shaped by genetic drift, recombination, and selection (Runemark et al., 2019). This genic view of introgression illustrates that the dichotomy between the deleterious and beneficial effects of introgression on the population level becomes more nuanced on the genomic level (Wu, 2001). Both effects can be acting simultaneously within a collection of genomes: some genomic regions will be homogenized by (adaptive) introgression, while other regions will remain species‐specific due to strong negative selection against introgressed loci.

The majority of conservation genetic studies only quantified the level of introgression between interacting populations, but they did not assess which genomic loci are being exchanged or not. Genomic regions that contribute to reproductive isolation, either because they contain barrier loci or because they are involved in local adaptation, do not introgress and will thus preserve species integrity, even in the face of high levels of hybridization. A study only quantifying introgression might thus warn for genetic swamping or species collapse, even though species‐specific loci will prevent this from happening. For instance, extensive introgression between taiga bean goose (*Anser fabalis fabalis*) and tundra bean goose (*A. f. serrirostris*) resulted in a largely homogeneous genomic landscape, but a few genomic islands of differentiation seem to prevent these taxa from merging (Ottenburghs et al., 2020). However, if reproductive isolation mechanisms break down completely, such as the loss of species recognition between fish species due to eutrophication (Vonlanthen et al., 2012), the genomic barriers preventing species from merging have been broken and the underlying loci can also introgress. Hence, the threat of genetic swamping might increase and conservation measures will be warranted. It is thus important to investigate the functional role of introgressed regions. If the genes at the exchanged genomic locations are important in adaptation to rapidly changing environments (e.g., immune genes), a local increase in genetic diversity can provide the necessary adaptive potential to deal with these challenges (Chan et al., 2019; Derry et al., 2019). The development of more powerful techniques to detect introgression (Hibbins & Hahn, 2021) and the increasing completeness and better annotation of genome assemblies (Peona et al., 2018) suggest that these types of analyses will be possible for nonmodel organisms in the near future.

The genic view of introgression also becomes apparent in studies that documented the exchange of adaptive traits between taxa. In most cases, these traits can be traced back to one or a few genetic loci, such as polymorphisms in the gene *vkorc1* that causes resistance to rodenticides and introgressed between European and Algerian mouse populations (Song et al., 2011). Similarly, adaptive changes in coat color have been linked to particular introgressed loci in wolves (Anderson et al., 2009) and snowshoe hares (Jones et al., 2018). Examples of adaptive introgression in plants include the transfer of herbivore resistance traits between *Helianthus* sunflowers (Whitney et al., 2006) and regulatory genes for certain ecological traits between *Senecio* plants (Kim et al., 2008). Two recent studies on anthropogenic hybridization documented adaptive introgression. Oziolor et al., (2019) showed that Gulf killifish (*Fundulus grandis*) adapted to high levels of water pollution through introgressive hybridization with the non‐native Atlantic killifish (*F. heteroclitus*). And a study on hybridization between the local moth species *Helicoverpa zea* and the invasive *H. armigera* revealed that an insecticide‐resistant locus introgressed into the local species and increased in frequency (Valencia‐Montoya et al., 2020). Interestingly, both studies relied on whole genome sequencing data, suggesting that it is difficult to detect adaptive introgression with less powerful sequencing methods. However, the methods for detecting adaptive introgression are improving (Moest et al., 2020; Setter et al., 2020; Zhang et al., 2020) and it will only be a matter of time before these methods can be applied more widely, as exemplified by the increasing availability of test statistics to detect introgression (Hibbins & Hahn, 2021). However, not intervening in anthropogenic hybridization events to provide the opportunity for adaptive introgression is generally not advisable and requires a thorough understanding of the study system (Allendorf et al., 2001). In summary, it is not only important to quantify how much genetic material introgressed, but also what has been exchanged.

## 7 INSIGHTS FROM PRE‐ANTHROPOCENE HYBRIDIZATION EVENTS?

Several anthropogenic hybridization events occur between species that have previously not overlapped in range. For example, hybridization between *Corbicula* clams in the European river Rhine probably started with the introduction of American lineages through cargo ships (Pfenninger et al., 2002). When two previously allopatric taxa interact for the first time prezygotic isolation mechanisms are often not well developed, resulting in high level of interbreeding and possibly introgression. Indeed, reproductive isolation is often weaker between allopatric taxa compared to sympatric taxa (Coyne & Orr, 1989; Presgraves, 2002), probably because reproductive isolation between sympatric taxa has been strengthened by reinforcement (Calabrese & Pfennig, 2020; Coughlan & Matute, 2020). Alternatively, some hybridizing taxa might have a history of hybridization with previous periods of secondary contact. For instance, polar bears and grizzly bears, that are currently interbreeding in the Arctic due to recent climate change (Kelly et al., 2010), have an extensive history of pre‐Anthropocene hybridization events (Kumar et al., 2017; Kutschera et al., 2014). This raises the question whether conservationists can apply insights from these past hybridization events to inform current policy.

One could compare past introgression patterns with the current dynamics of the human‐mediated hybridization event, potentially allowing researchers to assess different outcomes with greater confidence. For example, according to one theoretical model of genome evolution during speciation, genomic islands of differentiation that house barrier loci are expected to increase in size as genomic regions are linked together. This process—known as genome hitchhiking—leads to an average genome‐wide reduction in introgression, ultimately culminating in complete reproductive isolation (Feder et al., 2012; Flaxman et al., 2013). Comparing the genomic landscape between pre‐ and post‐Anthropocene hybridization events might reveal this expansion of genomic islands over time, suggesting that reproductive isolation has been strengthened and genetic swamping is less likely to occur. It is also possible that some taxa are in merging‐and‐diverging cycles where periods of geographic isolation are punctuated by introgression events (Grant & Grant, 2008; McKay & Zink, 2015). During each allopatric phase, more genetic divergence builds up between the taxa, resulting in lower levels of introgression during the subsequent merging phase. Human‐mediated changes might have sped up the occurrence of a merging event, such as the interbreeding of polar bears and grizzly bears. Reconstructing the dynamics during these merging‐and‐diverging cycles might provide insights into the likelihood of genetic swamping or species collapse during the anthropogenic introgression event. However, the environmental and genetic context of pre‐Anthropocene hybridization events might not be comparable with the dynamics in current hybrid zones (Gompert et al., 2017). To my knowledge, no study has explicitly compared pre‐Anthropocene hybridization events with current human‐induced cases. This knowledge gap provides an exciting avenue for further research that could lead to better conservation measures.

## 8 RETICULATION AND CONSERVATION

The genetic legacy of past hybridization events is still detectable in present‐day genomes (Lombal et al., 2020; Ottenburghs, 2020; Taylor & Larson, 2019). These genetic patterns can be especially apparent in phylogenomic studies where phylogenetic analyses of different genomic regions point to distinct evolutionary histories—as one would expect based on the genic view of introgression (Edelman et al., 2019; Li et al., 2019; Ottenburghs, Kraus, et al., 2017; Ottenburghs, Megens, et al., 2017). For instance, phylogenomic analyses of the cat family (Felidae) revealed that the phylogenetic signal for the species tree was concentrated within genomic regions of low recombination, whereas regions of high recombination were heavily influenced by past introgression (Li et al., 2019). Apart from introgressive hybridization, this phylogenetic incongruence between different genomic regions—known as gene tree discordance—can also be the outcome of other evolutionary processes, such as incomplete lineage sorting or gene duplications (Degnan & Rosenberg, 2009; Maddison, 1997). Several methods have been developed to discriminate between these processes and to deal with high levels phylogenetic incongruence (Kapli et al., 2020; Ottenburghs, Kraus, et al., 2017; Ottenburghs, Megens, et al., 2017; Zhou et al., 2020). One promising approach is the application of phylogenetic networks to highlight the reticulated nature of evolution (Blair & Ané, 2019; Ottenburghs et al., 2016).

Conservation efforts are still mainly focused on a tree‐like pattern of evolution in which distinct evolutionary lineages warrant protection. This perspective is reflected in the Endangered Species Act (ESA) in the United States which lists and protects vulnerable species and subspecies of plants and animals (Ellstrand et al., 2010). At the moment, interspecific hybrids are generally denied protection under ESA (although some hybrid plant species might be considered). Similarly, the IUCN Red list does not consider hybrids (IUCN, 2020). Other organizations do take hybrids into account or provide guidelines on how to deal with hybridization (Jackiw et al., 2015). For example, the Convention on International Trade in Endangered Species of Wild Fauna and Flora (CITES) has included some hybrids in Appendices I and II (i.e., species threatened with extinction or species that will become threatened without controlling trade). These examples clearly indicate that there are still large discrepancies in conservation efforts regarding hybrids between different countries and organizations. Acknowledging the reticulated nature of the evolutionary process by reconstructing past hybridization events and adopting a phylogenetic network approach can help to design better guidelines for hybrids (vonHoldt et al., 2018).

**FIGURE 3:**
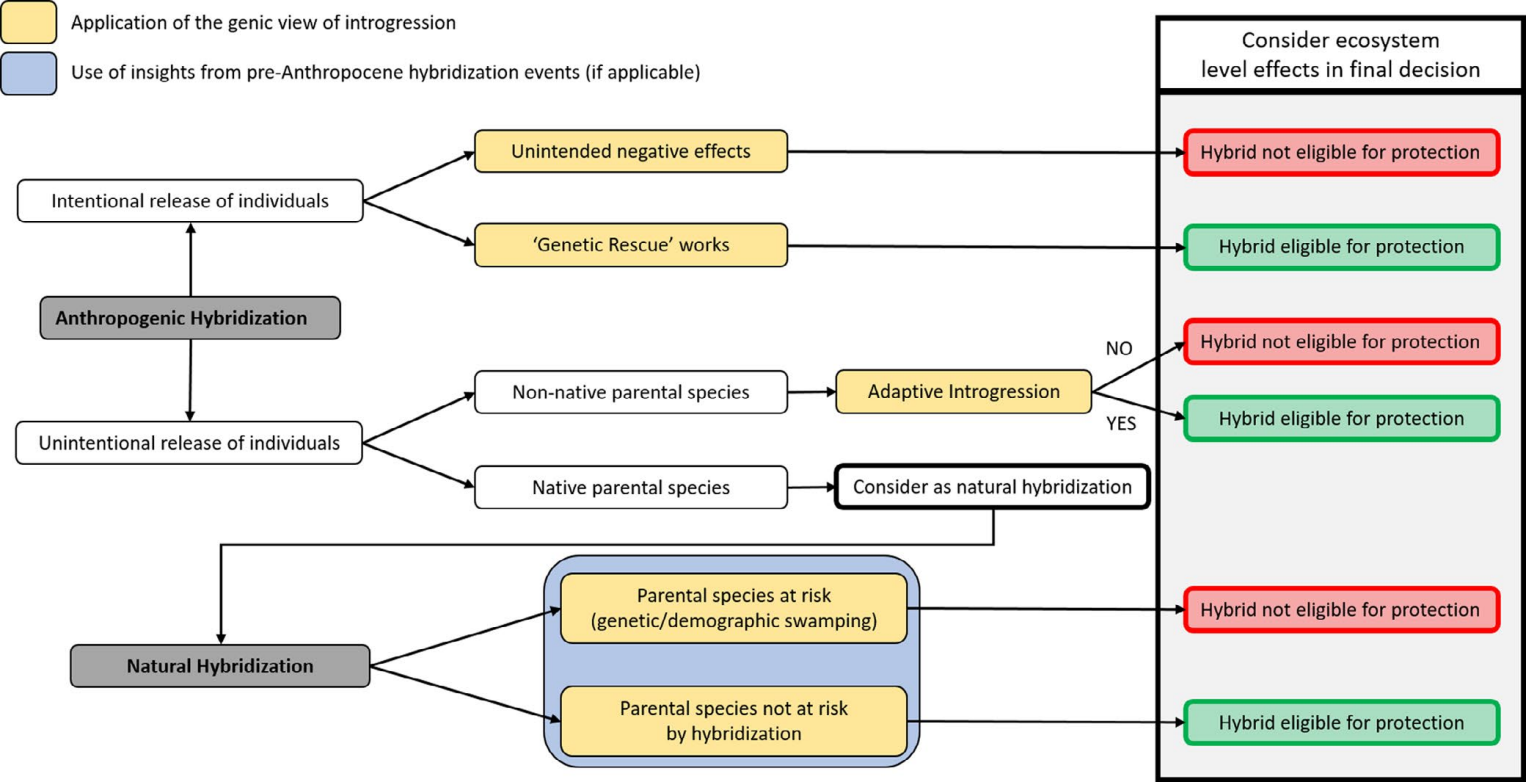
A decision tree to guide conservationists in determining the legal status of hybrids (based on Jackiw et al., 2015). Insights from speciation genomics (yellow) and past hybridization (blue) events are especially relevant in determining the risk for parental populations and the likelihood of adaptive introgression. In the final legal decision, it is important to also take into account potential ecosystem‐level effects of the hybrids. Please note that this decision tree only provides a rough framework and each hybridization event—anthropogenic or natural—should be assessed individually

Jackiw et al., (2015) proposed an elaborate framework to implement conservation efforts for hybrids, taking into account ethical and ecological considerations. We can incorporate insights from speciation genomics into this framework to deal with hybridization events in the Anthropocene (Figure 3). First, consider human‐mediated hybridization events (e.g., due to land‐use changes or the release of particular species). The release of individuals can be intentional as part of a genetic rescue program. If this program is closely monitored and there are no negative effects of hybridization, the hybrids are eligible for legal protection (Frankham, 2015). However, if the genetic rescue program leads to unforeseen issues, such as outbreeding depression or maladaptive introgression, and the hybrids threaten the endangered population, conservation efforts are needed (Frankham et al., 2011; Mills & Allendorf, 1996). This scenario also applies to the intentional release of game birds or fish that might negatively affect local populations through interbreeding (Randi, 2008). When the introduction of a particular species is unintentional—either due to individuals escaping from captivity or the spread of populations due to land‐use changes—the legal status of the hybrids depends on the native distribution of hybridizing species. If one parental species is non‐native, the hybrids cannot be legally protected and conservation measures should be implemented. For example, the introduction of North American *Corbicula* clams in the European river Rhine (Pfenninger et al., 2002). However, some studies documented adaptive introgression between a native and an exotic species (Oziolor et al., 2019; Valencia‐Montoya et al., 2020), indicating this possibility should be taken into account when setting the legal status of hybrids. When both parental species are native, the hybridization event can be treated as a natural phenomenon and the conservation status of the interbreeding species should be taken into account. If one or both species are threatened by genetic or demographic swamping, then legal protection of the resulting hybrids is not advisable and conservation efforts should be implemented. If the parental species are not threatened by hybridization, the situation needs to be assessed to determine whether hybridization could be beneficial (e.g., adaptive introgression or increasing genetic diversity). Here, insights from speciation genomics can be useful. However, be aware that this decision tree only provides a rough framework and each hybridization event—anthropogenic or natural—should be assessed individually (Allendorf et al., 2001).

An aspect that is currently missing from this framework is how anthropogenic hybridization affects ecosystem functioning. Most research on hybridization has focused on the interactions and genetic consequences between two or several taxa (Ottenburghs, 2019; Schwenk et al., 2008; Taylor & Larson, 2019). However, the production of hybrids not only affects the interbreeding taxa, it can also have far‐reaching consequences on an ecosystem level, altering food webs or nutrient cycles (Brennan et al., 2014). Some of these consequences are clearly negative, such as the spread of invasive pests (Corrêa et al., 2019) and the emergence of novel pathogens (Stukenbrock, 2016). But hybrids can also have a positive effect on other species in the ecosystem. For example, the spread of invasive *Spartina* hybrids provided extra habitat for the endangered California Ridgway's Rail (*Rallus obsoletus*) in the San Francisco Estuary (Ort & Thornton, 2016). Should the positive effect of these hybrids be taken into account in conservation efforts? The ecosystem perspective on anthropogenic hybridization adds another layer of complexity to the implementation of conservation measures.

## 9 CONCLUSION

As humans continue to change the environment and alter species distributions, more anthropogenic hybridization events will definitely occur. This will pose challenges for the conservation of endangered species, but also provide unique opportunities for evolutionary biologists. The interplay between conservation genomics and speciation genomics provides an exciting avenue for further research to gain important insights into the origin of species and how to protect them. In this review, I have illustrated this exchange of ideas by showing how insights from speciation genomics can guide the management of anthropogenic hybridization events. These insights range from practical issues (e.g., the development of diagnostic markers) to theoretical considerations, such as patterns of introgression, ancient hybridization events, and the reticulated nature of evolution.

From a conservation perspective, the unpredictability of (adaptive) introgression dynamics (Taylor & Larson, 2019) in combination with the strong link between anthropogenic hybridization and extinction risk (Todesco et al., 2016) suggests that the default course of action for human‐induced hybridization events should be the implementation of conservation measures to prevent hybridization. However, some studies documented the exchange of adaptive traits between native and non‐native species (Oziolor et al., 2019; Valencia‐Montoya et al., 2020), indicating that anthropogenic hybridization can result in adaptive introgression. The removal of introduced species and hybrids might have prevented the local species from adapting to the changing environment. These examples illustrate that each case of human‐induced hybridization should be judged separately (Allendorf et al., 2001). In addition, speciation genomic studies have shown that species can remain distinct in the face of high levels of introgression when species‐specific loci are not exchanged. Hence, high levels of introgression do not necessarily imply genetic swamping. It is not only important to quantify how much genetic material introgressed, but also what genomic regions have been exchanged. Finally, comparing current introgression dynamics with pre‐Anthropocene hybridization events might lead to novel insights on how to manage human‐mediated hybridization events, although this idea remains to be tested. Taking all these perspectives into account, in combination with the conservation status of the hybridizing taxa, thoughtful and evidence‐based conservation measures can be implemented.

## CONFLICT OF INTEREST

None declared.

## Data Availability

There are not data associated with this manuscript, which is solely based on literature.

## References

[eva13223-bib-0001] Alexander, D. , Novembre, J. , & Lange, K. (2009). Fast model‐based estimation of ancestry in unrelated individuals. Genome Research, 19(9), 1655–1664. 19648217 10.1101/gr.094052.109 PMC2752134

[eva13223-bib-0002] Alexander, T. J. , Vonlanthen, P. , & Seehausen, O. (2017). Does eutrophication‐driven evolution change aquatic ecosystems? Philosophical Transactions of the Royal Society B: Biological Sciences, 372(1712), 20160041. 10.1098/rstb.2016.0041 PMC5182437 27920386

[eva13223-bib-0003] Allendorf, F. W. , Leary, R. F. , Spruell, P. , & Wenburg, J. K. (2001). The problems with hybrids: Setting conservation guidelines. Trends in Ecology and Evolution, 16(11), 613–622. 10.1016/S0169-5347(01)02290-X

[eva13223-bib-0004] Alric, B. , Möst, M. , Domaizon, I. , Pignol, C. , Spaak, P. , & Perga, M.‐E. (2016). Local human pressures influence gene flow in a hybridizing *Daphnia* species complex. Journal of Evolutionary Biology, 29(4), 720–735. 10.1111/jeb.12820 26717418

[eva13223-bib-0005] Anderson, D. , Toma, R. , Negishi, Y. , Okuda, K. , Ishiniwa, H. , Hinton, T. G. , Nanba, K. , Tamate, H. B. , & Kaneko, S. (2019). Mating of escaped domestic pigs with wild boar and possibility of their offspring migration after the Fukushima Daiichi Nuclear Power Plant accident. Scientific Reports, 9(1), 1–6. 10.1038/s41598-019-47982-z 31395920 PMC6687819

[eva13223-bib-0006] Anderson, E. (1948). Hybridization of the habitat. Evolution, 2, 1–9.

[eva13223-bib-0007] Anderson, E. C. (2008). Bayesian inference of species hybrids using multilocus dominant genetic markers. Philosophical Transactions of the Royal Society B: Biological Sciences, 363(1505), 2841–2850. 10.1098/rstb.2008.0043 PMC2606736 18508754

[eva13223-bib-0008] Anderson, T. M. , vonHoldt, B. M. , Candille, S. I. , Musiani, M. , Greco, C. , Stahler, D. R. , Smith, D. W. , Padhukasahasram, B. , Randi, E. , Leonard, J. A. , Bustamante, C. D. , Ostrander, E. A. , Tang, H. , Wayne, R. K. , & Barsh, G. S. (2009). Molecular and evolutionary history of melanism in North American gray wolves. Science, 323(5919), 1339–1343. 10.1126/science.1165448 19197024 PMC2903542

[eva13223-bib-0009] Andrews, K. R. , Good, J. M. , Miller, M. R. , Luikart, G. , & Hohenlohe, P. A. (2016). Harnessing the power of RADseq for ecological and evolutionary genomics. Nature Reviews Genetics, 17(2), 81–92. 10.1038/nrg.2015.28 PMC4823021 26729255

[eva13223-bib-0010] Arnold, B. , Corbett‐Detig, R. B. , Hartl, D. , & Bomblies, K. (2013). RADseq underestimates diversity and introduces genealogical biases due to nonrandom haplotype sampling. Molecular Ecology, 22(11), 3179–3190. 10.1111/mec.12276 23551379

[eva13223-bib-0011] Arnold, M. (1997). Natural hybridization and evolution. Oxford University Press.

[eva13223-bib-0012] Arnold, M. , Ballerini, E. , & Brothers, A. (2012). Hybrid fitness, adaptation and evolutionary diversification: Lessons learned from Louisiana Irises. Heredity, 108(3), 159–166. 10.1038/hdy.2011.65 21792222 PMC3282389

[eva13223-bib-0013] Arnold, M. L. , & Kunte, K. (2017). Adaptive genetic exchange: A tangled history of admixture and evolutionary innovation. Trends in Ecology & Evolution, 32(8), 601–611. 10.1016/J.TREE.2017.05.007 28645486

[eva13223-bib-0014] Arnold, M. , & Martin, N. (2010). Hybrid fitness across time and habitats. Trends in Ecology and Evolution, 25(9), 530–536. 10.1016/j.tree.2010.06.005 20598770

[eva13223-bib-0015] Bangs, M. R. , Douglas, M. R. , Thompson, P. , & Douglas, M. E. (2017). Anthropogenic impacts facilitate native fish hybridization in the Bonneville Basin of western North America. Transactions of the American Fisheries Society, 146(1), 16–21. 10.1080/00028487.2016.1235611

[eva13223-bib-0016] Bangs, M. R. , Oswald, K. J. , Greig, T. W. , Leitner, J. K. , Rankin, D. M. , & Quattro, J. M. (2018). Introgressive hybridization and species turnover in reservoirs: A case study involving endemic and invasive basses (Centrarchidae: Micropterus) in southeastern North America. Conservation Genetics, 19(1), 57–69. 10.1007/s10592-017-1018-7

[eva13223-bib-0017] Battey, C. J. (2019). Evidence of linked selection on the z chromosome of hybridizing hummingbirds. Evolution, 74(4), 725–739. 10.1111/evo.13888 31859363

[eva13223-bib-0018] Baveja, P. , Tang, Q. , Lee, J. G. H. , & Rheindt, F. E. (2019). Impact of genomic leakage on the conservation of the endangered Milky Stork. Biological Conservation, 229, 59–66. 10.1016/J.BIOCON.2018.11.009

[eva13223-bib-0019] Bazykin, A. D. (1969). Hypothetical mechanism of speciation. Evolution, 23, 685–687. 28562864 10.1111/j.1558-5646.1969.tb03550.x

[eva13223-bib-0020] Beheregaray, L. B. , Pfeiffer, L. V. , Attard, C. R. M. , Sandoval‐Castillo, J. , Domingos, F. M. C. B. , Faulks, L. K. , Gilligan, D. M. , & Unmack, P. J. (2017). Genome‐wide data delimits multiple climate‐determined species ranges in a widespread Australian fish, the golden perch (*Macquaria ambigua*). Molecular Phylogenetics and Evolution, 111, 65–75. 10.1016/j.ympev.2017.03.021 28347889

[eva13223-bib-0021] Bell, R. C. , & Irian, C. G. (2019). Phenotypic and genetic divergence in reed frogs across a mosaic hybrid zone on São Tomé Island. Biological Journal of the Linnean Society, 128(3), 672–680. 10.1093/biolinnean/blz131

[eva13223-bib-0022] Blair, C. , & Ané, C. (2019). Phylogenetic trees and networks can serve as powerful and complementary approaches for analysis of genomic data. Systematic Biology, 69(3), 593–601. 10.1093/sysbio/syz056 31432090

[eva13223-bib-0023] Boecklen, W. J. , & Howard, D. J. (1997). Genetic analysis of hybrid zones: Numbers of markers and power of resolution. Ecology, 78(8), 2611–2616.

[eva13223-bib-0024] Bourgeois, Y. X. , Bertrand, J. A. , Delahaie, B. , Holota, H. , Thébaud, C. , & Milá, B. (2020). Differential divergence in autosomes and sex chromosomes is associated with intra‐island diversification at a very small spatial scale in a songbird lineage. Molecular Ecology, 29(6), 1137–1153. 10.1111/mec.15396 32107807

[eva13223-bib-0025] Bourke, P. M. , Voorrips, R. E. , Visser, R. G. F. , & Maliepaard, C. (2018). Tools for genetic studies in experimental populations of polyploids. Frontiers in Plant Science, 9, 513. 10.3389/fpls.2018.00513 29720992 PMC5915555

[eva13223-bib-0026] Brennan, A. C. , Woodward, G. , Seehausen, O. , Muñoz‐Fuentes, V. , Moritz, C. , Guelmami, A. , Abbott, R. J. , & Edelaar, P. (2014). Hybridization due to changing species distributions: Adding problems or solutions to conservation of biodiversity during global change? Evolutionary Ecology Research, 16(6), 475–491.

[eva13223-bib-0027] Bruce, S. A. , Kutsumi, Y. , Van Maaren, C. , & Hare, M. P. (2020). Stocked‐fish introgression into wild brook trout populations depends on habitat. Transactions of the American Fisheries Society, 149(4), 427–442. 10.1002/tafs.10239

[eva13223-bib-0028] Bruni, I. , De Mattia, F. , Fluch, S. , Ferrari, C. , Corazza, M. , Dinelli, E. , & Labra, M. (2016). Genetic introgression of hybrid Rhododendron x intermedium Tausch is habitat mediated: Evidences from south‐eastern Alps (Italy). Plant Biosystems, 150(3), 449–458. 10.1080/11263504.2014.986246

[eva13223-bib-0029] Burri, R. (2017). Dissecting differentiation landscapes: A linked selection's perspective. Journal of Evolutionary Biology, 30(8), 1501–1505. 10.1111/jeb.13108 28786187

[eva13223-bib-0030] Calabrese, G. M. , & Pfennig, K. S. (2020). Reinforcement and the proliferation of species. Journal of Heredity, 111(1), 138–146. 10.1093/jhered/esz073 31850499

[eva13223-bib-0031] Campbell, C. R. , Poelstra, J. W. , & Yoder, A. D. (2018). What is Speciation Genomics? The roles of ecology, gene flow, and genomic architecture in the formation of species. Biological Journal of the Linnean Society, 124(4), 561–583.

[eva13223-bib-0032] Canavan, K. , Paterson, I. D. , Lambertini, C. , & Hill, M. P. (2018). Expansive reed populations—Alien invasion or disturbed wetlands? AoB Plants, 10(2), ply014. 10.1093/aobpla/ply014 29593854 PMC5861408

[eva13223-bib-0033] Carantón‐Ayala, D. , Avendaño, J. E. , & Cadena, C. D. (2018). Hybridization in brushfinches (Atlapetes, Emberizidae) from the southeast Andes of Colombia: A consequence of habitat disturbance? Journal of Ornithology, 159(3), 713–722. 10.1007/s10336-018-1544-1

[eva13223-bib-0034] Carney, S. E. , Gardner, K. A. , & Rieseberg, L. H. (2000). Evolutionary changes over the fifty‐year history of a hybrid population of sunflowers (Helianthus). Evolution, 54(2), 462–474. 10.1111/j.0014-3820.2000.tb00049.x 10937223

[eva13223-bib-0035] Chafin, T. K. , Douglas, M. R. , Martin, B. T. , & Douglas, M. E. (2019). Hybridization drives genetic erosion in sympatric desert fishes of western North America. Heredity, 123(6), 759–773. 10.1038/s41437-019-0259-2 31431737 PMC6834602

[eva13223-bib-0036] Chan, W. Y. , Hoffmann, A. A. , & Oppen, M. J. H. (2019). Hybridization as a conservation management tool. Conservation Letters, 12(5), e12652. 10.1111/conl.12652

[eva13223-bib-0037] Chunco, A. J. (2014). Hybridization in a warmer world. Ecology and Evolution, 4(10), 2019–2031. 10.1002/ece3.1052 24963394 PMC4063493

[eva13223-bib-0038] Clark, L. V. , Lipka, A. E. , & Sacks, E. J. (2019). polyRAD: Genotype calling with uncertainty from sequencing data in polyploids and diploids. G3: Genes, Genomes, Genetics, 9(3), 663–673. 10.1534/g3.118.200913 30655271 PMC6404598

[eva13223-bib-0039] Coppi, A. , Baker, A. J. M. , Bettarini, I. , Colzi, I. , Echevarria, G. , Pazzagli, L. , Gonnelli, C. , & Selvi, F. (2020). Population Genetics of Odontarrhena (Brassicaceae) from Albania: The effects of anthropic habitat disturbance, soil, and altitude on a Ni‐Hyperaccumulator plant group from a major serpentine hotspot. Plants, 9(12), 1686. 10.3390/plants9121686 PMC7759883 33271845

[eva13223-bib-0040] Corrêa, A. S. , Cordeiro, E. M. , & Omoto, C. (2019). Agricultural insect hybridization and implications for pest management. Pest Management Science, 75(11), 2857–2864. 10.1002/ps.5495 31124266

[eva13223-bib-0041] Coughlan, J. M. , & Matute, D. R. (2020). The importance of intrinsic postzygotic barriers throughout the speciation process. Philosophical Transactions of the Royal Society of London. Series B, Biological Sciences, 375(1806), 20190533. 10.1098/rstb.2019.0533 32654642 PMC7423280

[eva13223-bib-0042] Coyne, J. A. , & Orr, H. A. (1989). Patterns of speciation in Drosophila. Evolution, 43(2), 362–381. 10.1111/j.1558-5646.1989.tb04233.x 28568554

[eva13223-bib-0043] Crispo, E. , Moore, J. S. , Lee‐Yaw, J. A. , Gray, S. M. , & Haller, B. C. (2011). Broken barriers: Human‐induced changes to gene flow and introgression in animals: An examination of the ways in which humans increase genetic exchange among populations and species and the consequences for biodiversity. BioEssays, 33(7), 508–518. 10.1002/bies.201000154 21523794

[eva13223-bib-0044] Crutzen, P. J. (2002). Geology of mankind. Nature, 415(6867), 23. 10.1038/415023a 11780095

[eva13223-bib-0045] Degnan, J. H. , & Rosenberg, N. A. (2009). Gene tree discordance, phylogenetic inference and the multispecies coalescent. Trends in Ecology & Evolution, 24(6), 332–340. 10.1016/J.TREE.2009.01.009 19307040

[eva13223-bib-0046] Derry, A. M. , Fraser, D. J. , Brady, S. P. , Astorg, L. , Lawrence, E. R. , Martin, G. K. , Matte, J. M. , Negrín Dastis, J. O. , Paccard, A. , Barrett, R. D. H. , Chapman, L. J. , Lane, J. E. , Ballas, C. G. , Close, M. , & Crispo, E. (2019). Conservation through the lens of (mal)adaptation: Concepts and meta‐analysis. Evolutionary Applications, 12(7), 1287–1304. 10.1111/eva.12791 31417615 PMC6691223

[eva13223-bib-0047] Dubey, S. , Lavanchy, G. , Thiebaud, J. , & Dufresnes, C. (2019). Herps without borders: A new newt case and a review of transalpine alien introductions in western Europe. Amphibia Reptilia, 40(1), 13–27. 10.1163/15685381-20181028

[eva13223-bib-0048] Dufresnes, C. , Leuenberger, J. , Amrhein, V. , Bühler, C. , Thiébaud, J. , Bohnenstengel, T. , & Dubey, S. (2018). Invasion genetics of marsh frogs (*Pelophylax ridibundus* sensu lato) in Switzerland. Biological Journal of the Linnean Society, 123(2), 402–410. 10.1093/biolinnean/blx140

[eva13223-bib-0049] Edelman, N. B. , Frandsen, P. B. , Miyagi, M. , Clavijo, B. , Davey, J. , Dikow, R. B. , García‐Accinelli, G. , Van Belleghem, S. M. , Patterson, N. , Neafsey, D. E. , Challis, R. , Kumar, S. , Moreira, G. R. P. , Salazar, C. , Chouteau, M. , Counterman, B. A. , Papa, R. , Blaxter, M. , Reed, R. D. , … Mallet, J. (2019). Genomic architecture and introgression shape a butterfly radiation. Science, 366(6465), 594–599. 10.1126/science.aaw2090 31672890 PMC7197882

[eva13223-bib-0050] Edmands, S. (1999). Heterosis and outbreeding depression in interpopulation crosses spanning a wide range of divergence. Evolution, 53(6), 1757–1768. 10.1111/j.1558-5646.1999.tb04560.x 28565458

[eva13223-bib-0051] Ellstrand, N. C. , Biggs, D. , Kaus, A. , Lubinsky, P. , McDade, L. A. , Preston, K. , Prince, L. M. , Regan, H. M. , Rorive, V. , Ryder, O. A. , & Schierenbeck, K. A. (2010). Got hybridization? A multidisciplinary approach for informing science policy. BioScience, 60(5), 384–388. 10.1525/bio.2010.60.5.8

[eva13223-bib-0052] Escalante, M. A. , Perrier, C. , García‐De León, F. J. , Ruiz‐Luna, A. , Ortega‐Abboud, E. , & Manel, S. (2020). Genotyping‐by‐sequencing reveals the effects of riverscape, climate and interspecific introgression on the genetic diversity and local adaptation of the endangered Mexican golden trout (*Oncorhynchus chrysogaster*). Conservation Genetics, 21(5), 907–926. 10.1007/s10592-020-01297-z

[eva13223-bib-0053] Fadakar, D. , Malekian, M. , Hemami, M. R. , Lerp, H. , Rezaei, H. R. , & Bärmann, E. V. (2020). Repeated hybridization of two closely related gazelle species (*Gazella bennettii* and *Gazella subgutturosa*) in central Iran. Ecology and Evolution, 10(20), 11372–11386. 10.1002/ece3.6774 33144971 PMC7593154

[eva13223-bib-0054] Faulks, L. , & Östman, Ö. (2016). Genetic Diversity and hybridisation between native and introduced Salmonidae fishes in a Swedish Alpine Lake. PLoS One, 11(3), e0152732. 10.1371/journal.pone.0152732 27032100 PMC4816307

[eva13223-bib-0055] Feder, J. L. , Egan, S. P. , & Nosil, P. (2012). The genomics of speciation‐with‐gene‐flow. Trends in Genetics, 28(7), 342–350. 22520730 10.1016/j.tig.2012.03.009

[eva13223-bib-0056] Feulner, P. G. D. , & Seehausen, O. (2019). Genomic insights into the vulnerability of sympatric whitefish species flocks. Molecular Ecology, 28(3), 615–629. 10.1111/mec.14977 30554444

[eva13223-bib-0057] Ficetola, G. F. , Lunghi, E. , Cimmaruta, R. , & Manenti, R. (2019). Transgressive niche across a salamander hybrid zone revealed by microhabitat analyses. Journal of Biogeography, 46(7), jbi.13621. 10.1111/jbi.13621

[eva13223-bib-0058] Fisher, H. S. , Wong, B. B. , & Rosenthal, G. G. (2006). Alteration of the chemical environment disrupts communication in a freshwater fish. Proceedings of the Royal Society B: Biological Sciences, 273(1591), 1187–1193. 10.1098/rspb.2005.3406 PMC1560282 16720390

[eva13223-bib-0059] Flaxman, S. M. , Feder, J. L. , & Nosil, P. (2013). Genetic hitchhiking and the dynamic buildup of genomic divergence during speciation with gene flow. Evolution, 67(9), 2577–2591. 10.1111/evo.12055 24033168

[eva13223-bib-0060] Forcina, G. , Guerrini, M. , & Barbanera, F. (2020). Non‐native and hybrid in a changing environment: Conservation perspectives for the last Italian red‐legged partridge (*Alectoris rufa*) population with long natural history. Zoology, 138, 125740. 10.1016/j.zool.2019.125740 31935616

[eva13223-bib-0061] Forcina, G. , Guerrini, M. , Khaliq, I. , Khan, A. A. , & Barbanera, F. (2018). Human‐modified biogeographic patterns and conservation in game birds: The dilemma of the black francolin (Francolinus francolinus, Phasianidae) in Pakistan. PLoS One, 13(10), e0205059. 10.1371/journal.pone.0205059 30289901 PMC6173408

[eva13223-bib-0062] Frankham, R. (2015). Genetic rescue of small inbred populations: Meta‐analysis reveals large and consistent benefits of gene flow. Molecular Ecology, 24(11), 2610–2618. 10.1111/mec.13139 25740414

[eva13223-bib-0063] Frankham, R. , Ballou, J. D. , Eldridge, M. D. B. , Lacy, R. C. , Ralls, K. , Dudash, M. R. , & Fenster, C. B. (2011). Predicting the probability of outbreeding depression. Conservation Biology, 25(3), 465–475. 10.1111/j.1523-1739.2011.01662.x 21486369

[eva13223-bib-0064] Fraser, G. S. , DeHaan, P. W. , Smith, C. T. , Von Bargen, J. F. , Cooper, M. R. , & Desgrosseillier, T. J. (2020). Overlap of spatial and temporal spawning distributions of spring and summer Chinook Salmon results in hybridization in the upper Columbia River. Transactions of the American Fisheries Society, 149(5), 517–531. 10.1002/tafs.10258

[eva13223-bib-0065] Funk, W. C. , Forester, B. R. , Converse, S. J. , Darst, C. , & Morey, S. (2019). Improving conservation policy with genomics: A guide to integrating adaptive potential into U.S. Endangered Species Act decisions for conservation practitioners and geneticists. Conservation Genetics, 20(1), 115–134. 10.1007/s10592-018-1096-1

[eva13223-bib-0066] Galaverni, M. , Caniglia, R. , Pagani, L. , Fabbri, E. , Boattini, A. , & Randi, E. (2017). Disentangling timing of admixture, patterns of introgression, and phenotypic indicators in a hybridizing wolf population. Molecular Biology and Evolution, 34(9), 2324–2339. 28549194 10.1093/molbev/msx169 PMC5850710

[eva13223-bib-0067] Garmendia, A. , Merle, H. , Ruiz, P. , & Ferriol, M. (2018). Distribution and ecological segregation on regional and microgeographic scales of the diploid *Centaurea aspera* L., the tetraploid *C. seridis* L., and their triploid hybrids (Compositae). PeerJ, 2018(7), e5209. 10.7717/peerj.5209 PMC6034602 30002989

[eva13223-bib-0068] Garroway, C. J. , Bowman, J. , Cascaden, T. J. , Holloway, G. L. , Mahan, C. G. , Malcolm, J. R. , Steele, M. A. , Turner, G. , & Wilson, P. J. (2010). Climate change induced hybridization in flying squirrels. Global Change Biology, 16(1), 113–121. 10.1111/j.1365-2486.2009.01948.x

[eva13223-bib-0069] Gibson, I. , Welsh, A. B. , Welsh, S. A. , & Cincotta, D. A. (2019). Genetic swamping and possible species collapse: Tracking introgression between the native Candy Darter and introduced Variegate Darter. Conservation Genetics, 20(2), 287–298. 10.1007/s10592-018-1131-2

[eva13223-bib-0070] Glasheen, P. M. , Burks, R. L. , Campos, S. R. , & Hayes, K. A. (2020). First evidence of introgressive hybridization of apple snails (*Pomacea* spp.) in their native range. Journal of Molluscan Studies, 86(2), 96–103. 10.1093/mollus/eyz035 PMC7182095 32362703

[eva13223-bib-0071] Gompert, Z. , Mandeville, E. G. , & Buerkle, C. A. (2017). Analysis of population genomic data from hybrid zones. Annual Review of Ecology, Evolution, and Systematics, 48, 207–229. 10.1146/annurev-ecolsys-110316-022652

[eva13223-bib-0072] Gong, S. , Vamberger, M. , Auer, M. , Praschag, P. , & Fritz, U. (2018). Millennium‐old farm breeding of Chinese softshell turtles (*Pelodiscus* spp.) results in massive erosion of biodiversity. The Science of Nature, 105(5–6), 1–10. 10.1007/s00114-018-1558-9 29728774

[eva13223-bib-0073] Grabenstein, K. C. , & Taylor, S. A. (2018). Breaking barriers: Causes, consequences, and experimental utility of human‐mediated hybridization. Trends in Ecology and Evolution, 33(3), 198–212. 10.1016/j.tree.2017.12.008 29306562

[eva13223-bib-0074] Graham, C. F. , Eberts, R. L. , Goncin, U. , & Somers, C. M. (2020). Spontaneous hybridization and introgression between walleye (*Sander vitreus*) and sauger (*Sander canadensis*) in two large reservoirs: Insights from genotyping by sequencing. Evolutionary Applications. 10.1111/eva.13174 PMC8061268 33897814

[eva13223-bib-0075] Grant, B. R. , & Grant, P. R. (2008). Fission and fusion of Darwin’s finches populations. Philosophical Transactions of the Royal Society of London. Series B, Biological Sciences, 363(1505), 2821–2829. 10.1098/rstb.2008.0051 18508750 PMC2606742

[eva13223-bib-0076] Grobler, P. , van Wyk, A. M. , Dalton, D. L. , van Vuuren, B. J. , & Kotzé, A. (2018). Assessing introgressive hybridization between blue wildebeest (*Connochaetes taurinus*) and black wildebeest (*Connochaetes gnou*) from South Africa. Conservation Genetics, 19(4), 981–993. 10.1007/s10592-018-1071-x

[eva13223-bib-0077] Guivier, E. , Gilles, A. , Pech, N. , Duflot, N. , Tissot, L. , & Chappaz, R. (2019). Canals as ecological corridors and hybridization zones for two cyprinid species. Hydrobiologia, 830(1), 1–16. 10.1007/s10750-018-3843-1

[eva13223-bib-0078] Harrisson, K. A. , Pavlova, A. , Gonçalves da Silva, A. , Rose, R. , Bull, J. K. , Lancaster, M. L. , Murray, N. , Quin, B. , Menkhorst, P. , Magrath, M. J. L. , & Sunnucks, P. (2016). Scope for genetic rescue of an endangered subspecies though re‐establishing natural gene flow with another subspecies. Molecular Ecology, 25(6), 1242–1258. 10.1111/mec.13547 26820991

[eva13223-bib-0079] Heber, S. , Varsani, A. , Kuhn, S. , Girg, A. , Kempenaers, B. , & Briskie, J. (2013). The genetic rescue of two bottlenecked South Island robin populations using translocations of inbred donors. Proceedings of the Royal Society B: Biological Sciences, 280(1752), 20122228. 10.1098/rspb.2012.2228 PMC3574298 23235701

[eva13223-bib-0080] Hedrick, P. W. (2013). Adaptive introgression in animals: Examples and comparison to new mutation and standing variation as sources of adaptive variation. Molecular Ecology, 22(18), 4606–4618. 10.1111/mec.12415 23906376

[eva13223-bib-0081] Hibbins, M. , & Hahn, M. (2021). Phylogenomic approaches to detecting and characterizing introgression. EcoEvoRxiv. 10.32942/OSF.IO/UAHD8 PMC9208645 34788444

[eva13223-bib-0082] Howell, P. J. (2018). Changes in native bull trout and non‐native brook trout distributions in the upper Powder River basin after 20 years, relationships to water temperature and implications of climate change. Ecology of Freshwater Fish, 27(3), 710–719. 10.1111/eff.12386

[eva13223-bib-0083] Huuskonen, H. , Shikano, T. , Mehtätalo, L. , Kettunen, J. , Eronen, R. , Toiviainen, A. , & Kekäläinen, J. (2017). Anthropogenic environmental changes induce introgression in sympatric whitefish ecotypes. Biological Journal of the Linnean Society, 121(3), 613–626. 10.1093/biolinnean/blx010

[eva13223-bib-0084] IUCN . (2020). IUCN Red List of Threatened Species. Retrieved from https://www.iucnredlist.org/resources/tax‐sources

[eva13223-bib-0085] Jackiw, R. N. , Mandil, G. , & Hager, H. A. (2015). A framework to guide the conservation of species hybrids based on ethical and ecological considerations. Conservation Biology, 29(4), 1040–1051. 10.1111/cobi.12526 25976359

[eva13223-bib-0086] Jones, M. R. , Mills, L. S. , Alves, P. C. , Callahan, C. M. , Alves, J. M. , Lafferty, D. J. R. , Jiggins, F. M. , Jensen, J. D. , Melo‐Ferreira, J. , & Good, J. M. (2018). Adaptive introgression underlies polymorphic seasonal camouflage in snowshoe hares. Science, 360(6395), 1355–1358. 10.1126/science.aar5273 29930138

[eva13223-bib-0087] Kapli, P. , Yang, Z. , & Telford, M. J. (2020). Phylogenetic tree building in the genomic age. Nature Reviews Genetics, 21, 428–444. 10.1038/s41576-020-0233-0 32424311

[eva13223-bib-0088] Kelly, B. P. , Whiteley, A. , & Tallmon, D. (2010). The Arctic melting pot. Nature, 468(7326), 891. 10.1038/468891a 21164461

[eva13223-bib-0089] Key, K. H. L. (1968). The concept of stasipatric speciation. Systematic Zoology, 17, 14–22.

[eva13223-bib-0090] Kim, M. , Cui, M.‐L. , Cubas, P. , Gillies, A. , Lee, K. , Chapman, M. A. , Abbott, R. J. , & Coen, E. (2008). Regulatory genes control a key morphological and ecological trait transferred between species. Science, 322(5904), 1116–1119. 10.1126/science.1164371 19008450

[eva13223-bib-0091] Kumar, V. , Lammers, F. , Bidon, T. , Pfenninger, M. , Kolter, L. , Nilsson, M. A. , & Janke, A. (2017). The evolutionary history of bears is characterized by gene flow across species. Scientific Reports, 7(1), 46487. 10.1038/srep46487 28422140 PMC5395953

[eva13223-bib-0092] Kutschera, V. E. , Bidon, T. , Hailer, F. , Rodi, J. L. , Fain, S. R. , & Janke, A. (2014). Bears in a forest of gene trees: Phylogenetic inference is complicated by incomplete lineage sorting and gene flow. Molecular Biology and Evolution, 31(8), 2004–2017. 10.1093/molbev/msu186 24903145 PMC4104321

[eva13223-bib-0093] Lamichhaney, S. , Han, F. , Webster, M. T. , Andersson, L. , Grant, B. R. , & Grant, P. R. (2018). Rapid hybrid speciation in Darwin’s finches. Science, 359(6372), 224–228. 10.1126/science.aao4593 29170277

[eva13223-bib-0094] Larson, E. L. , Tinghitella, R. M. , & Taylor, S. A. (2019). Insect hybridization and climate change. Frontiers in Ecology and Evolution, 7, 348. 10.3389/fevo.2019.00348

[eva13223-bib-0095] Lau, C. L. F. , & Jacobs, D. K. (2017). Introgression between ecologically distinct species following increased salinity in the Colorado Delta‐ Worldwide implications for impacted estuary diversity. PeerJ, 2017(12), e4056. 10.7717/peerj.4056 PMC5731342 29250463

[eva13223-bib-0096] Lavretsky, P. , Janzen, T. , & McCracken, K. G. (2019). Identifying hybrids & the genomics of hybridization: Mallards & American black ducks of Eastern North America. Ecology and Evolution, 9(6), 3470–3490. 10.1002/ece3.4981 30962906 PMC6434578

[eva13223-bib-0097] Lavretsky, P. , McInerney, N. R. , Mohl, J. E. , Brown, J. I. , James, H. F. , McCracken, K. G. , & Fleischer, R. C. (2020). Assessing changes in genomic divergence following a century of human mediated secondary contact among wild and captive‐bred ducks. Molecular Ecology, 29(3), 578–595. 10.1111/mec.15343 31872482

[eva13223-bib-0098] Lemopoulos, A. , Prokkola, J. M. , Uusi‐Heikkilä, S. , Vasemägi, A. , Huusko, A. , Hyvärinen, P. , Koljonen, M. L. , Koskiniemi, J. , & Vainikka, A. (2019). Comparing RADseq and microsatellites for estimating genetic diversity and relatedness — Implications for brown trout conservation. Ecology and Evolution, 9(4), 2106–2120. 10.1002/ece3.4905 30847096 PMC6392366

[eva13223-bib-0099] Lewis, S. L. , & Maslin, M. A. (2015). Defining the Anthropocene. Nature, 519(7542), 171–180. 10.1038/nature14258 25762280

[eva13223-bib-0100] Li, G. , Figueiró, H. V. , Eizirik, E. , & Murphy, W. J. (2019). Recombination‐aware phylogenomics reveals the structured genomic landscape of hybridizing cat species. Molecular Biology and Evolution, 36(10), 2111–2126. 10.1093/molbev/msz139 31198971 PMC6759079

[eva13223-bib-0101] Lombal, A. J. , O'dwyer, J. E. , Friesen, V. , Woehler, E. J. , & Burridge, C. P. (2020). Identifying mechanisms of genetic differentiation among populations in vagile species: Historical factors dominate genetic differentiation in seabirds. Biological Reviews, 95(3), 625–651. 10.1111/brv.12580 32022401

[eva13223-bib-0102] Lowry, D. B. , Hoban, S. , Kelley, J. L. , Lotterhos, K. E. , Reed, L. K. , Antolin, M. F. , & Storfer, A. (2016). Breaking RAD: An evaluation of the utility of restriction site associated DNA sequencing for genome scans of adaptation. Molecular Ecology Resources, 17(2), 142–152. 10.1111/1755-0998.12596 27860289 PMC5446919

[eva13223-bib-0103] Maddison, W. P. (1997). Gene trees in species trees. Systematic Biology, 46(3), 523–536. 10.1093/sysbio/46.3.523

[eva13223-bib-0104] Mallet, J. (2007). Hybrid speciation. Nature, 446(7133), 279–283. 10.1038/nature05706 17361174

[eva13223-bib-0105] Malukiewicz, J. (2019). A review of experimental, natural, and anthropogenic hybridization in Callithrix marmosets. International Journal of Primatology, 40(1), 72–98. 10.1007/s10764-018-0068-0

[eva13223-bib-0106] Mank, J. E. , Carlson, J. E. , & Brittingham, M. C. (2004). A century of hybridization: Decreasing genetic distance between American black ducks and mallards. Conservation Genetics, 5(3), 395–403. 10.1023/B:COGE.0000031139.55389.b1

[eva13223-bib-0107] Marques, D. A. , Meier, J. I. , & Seehausen, O. (2019). A combinatorial view on speciation and adaptive radiation. Trends in Ecology & Evolution, 34(6), 531–544.Retrieved from https://www.sciencedirect.com/science/article/pii/S0169534719300552 30885412 10.1016/j.tree.2019.02.008

[eva13223-bib-0108] Mayr, E. (1963). Animal species and evolution. Belknap Press of Harvard University Press.

[eva13223-bib-0109] McFarlane, S. E. , Hunter, D. C. , Senn, H. V. , Smith, S. L. , Holland, R. , Huisman, J. , & Pemberton, J. M. (2020). Increased genetic marker density reveals high levels of admixture between red deer and introduced Japanese sika in Kintyre. Scotland. Evolutionary Applications, 13(2), 432–441. 10.1111/eva.12880 31993087 PMC6976951

[eva13223-bib-0110] McFarlane, S. E. , & Pemberton, J. M. (2019). Detecting the true extent of introgression during anthropogenic hybridization. Trends in Ecology & Evolution, 34(4), 315–326. 10.1016/J.TREE.2018.12.013 30655011

[eva13223-bib-0111] McKay, B. D. , & Zink, R. M. (2015). Sisyphean evolution in Darwin’s finches. Biological Reviews, 90(3), 689–698. 10.1111/brv.12127 25040800

[eva13223-bib-0112] Miller, J. , Poissant, J. , Hogg, J. , & Coltman, D. (2012). Genomic consequences of genetic rescue in an insular population of bighorn sheep (*Ovis canadensis*). Molecular Ecology, 21(7), 1583–1596. 10.1111/j.1365-294X.2011.05427.x 22257293

[eva13223-bib-0113] Miller, S. M. , Moeller, C.‐H. , Harper, C. K. , & Bloomer, P. (2020). Anthropogenic movement results in hybridisation in impala in southern Africa. Conservation Genetics, 21, 653–663. 10.1007/s10592-020-01276-4

[eva13223-bib-0114] Millette, K. L. , Gonzalez, A. , & Cristescu, M. E. (2020). Breaking ecological barriers: Anthropogenic disturbance leads to habitat transitions, hybridization, and high genetic diversity. Science of the Total Environment, 740, 140046. 10.1016/j.scitotenv.2020.140046 32563876

[eva13223-bib-0115] Mills, L. S. , & Allendorf, F. W. (1996). The one‐migrant‐per‐generation rule in conservation and management. Conservation Biology, 10(6), 1509–1518. 10.1046/j.1523-1739.1996.10061509.x

[eva13223-bib-0116] Milot, E. , Béchet, A. , & Maris, V. (2020). The dimensions of evolutionary potential in biological conservation. Evolutionary Applications, 13(6), 1363–1379. 10.1111/eva.12995 32684964 PMC7359841

[eva13223-bib-0117] Moest, M. , Van Belleghem, S. M. , James, J. E. , Salazar, C. , Martin, S. H. , Barker, S. L. , Moreira, G. R. P. , Mérot, C. , Joron, M. , Nadeau, N. J. , Steiner, F. M. , & Jiggins, C. D. (2020). Selective sweeps on novel and introgressed variation shape mimicry loci in a butterfly adaptive radiation. PLOS Biology, 18(2), e3000597. 10.1371/journal.pbio.3000597 32027643 PMC7029882

[eva13223-bib-0118] Morais, P. , & Reichard, M. (2018). Cryptic invasions: A review. Science of the Total Environment, 613, 1438–1448. 10.1016/j.scitotenv.2017.06.133 28648374

[eva13223-bib-0119] Morii, K. , Nakano, M. , & Takakura, K. I. (2018). Does simultaneous and sympatric reproduction between two native spined loaches lead to reproductive interference and local extinction? Environmental Biology of Fishes, 101(9), 1407–1416. 10.1007/s10641-018-0787-2

[eva13223-bib-0120] Moulton, L. L. , Vallender, R. , Artuso, C. , & Koper, N. (2017). The final frontier: Early‐stage genetic introgression and hybrid habitat use in the northwestern extent of the Golden‐winged Warbler breeding range. Conservation Genetics, 18(6), 1481–1487. 10.1007/s10592-017-0989-8

[eva13223-bib-0121] Muhlfeld, C. C. , Kalinowski, S. T. , McMahon, T. E. , Taper, M. L. , Painter, S. , Leary, R. F. , & Allendorf, F. W. (2009). Hybridization rapidly reduces fitness of a native trout in the wild. Biology Letters, 5(3), 328–331. 10.1098/rsbl.2009.0033 19324629 PMC2679930

[eva13223-bib-0122] Muhlfeld, C. C. , Kovach, R. P. , Al‐Chokhachy, R. , Amish, S. J. , Kershner, J. L. , Leary, R. F. , Lowe, W. H. , Luikart, G. , Matson, P. , Schmetterling, D. A. , Shepard, B. B. , Westley, P. A. H. , Whited, D. , Whiteley, A. , & Allendorf, F. W. (2017). Legacy introductions and climatic variation explain spatiotemporal patterns of invasive hybridization in a native trout. Global Change Biology, 23(11), 4663–4674. 10.1111/gcb.13681 28374524

[eva13223-bib-0123] Nevado, B. , Harris, S. A. , Beaumont, M. A. , & Hiscock, S. J. (2020). Rapid homoploid hybrid speciation in British gardens: The origin of Oxford ragwort (*Senecio squalidus*). Molecular Ecology, 29(21), 4221–4233. 10.1111/mec.15630 32911573

[eva13223-bib-0124] O’Donnell, R. P. , Drost, C. A. , & Mock, K. E. (2017). Cryptic invasion of Northern Leopard Frogs (*Rana pipiens*) across phylogeographic boundaries and a dilemma for conservation of a declining amphibian. Biological Invasions, 19(3), 1039–1052. 10.1007/s10530-016-1320-1

[eva13223-bib-0125] Ort, B. S. , & Thornton, W. J. (2016). Changes in the population genetics of an invasive Spartina after 10 years of management. Biological Invasions, 18(8), 2267–2281. 10.1007/s10530-016-1177-3

[eva13223-bib-0126] Ortego, J. , Gugger, P. F. , & Sork, V. L. (2017). Impacts of human‐induced environmental disturbances on hybridization between two ecologically differentiated Californian oak species. New Phytologist, 213(2), 942–955. 10.1111/nph.14182 27621132

[eva13223-bib-0127] Oswald, J. A. , Harvey, M. G. , Remsen, R. C. , Foxworth, D. U. , Dittmann, D. L. , Cardiff, S. W. , & Brumfield, R. T. (2019). Evolutionary dynamics of hybridization and introgression following the recent colonization of Glossy Ibis (Aves: *Plegadis falcinellus*) into the New World. Molecular Ecology, 28(7), 1675–1691. 10.1111/MEC.15008 30614583

[eva13223-bib-0128] Ottenburghs, J. (2018). Exploring the hybrid speciation continuum in birds. Ecology and Evolution, 8(24), 13027–13034. 10.1002/ece3.4558 30619602 PMC6308868

[eva13223-bib-0129] Ottenburghs, J. (2019). Multispecies hybridization in birds. Avian Research, 10, 20. 10.1186/s40657-019-0159-4

[eva13223-bib-0130] Ottenburghs, J. (2020). Ghost introgression: Spooky Gene flow in the distant past. BioEssays, 42(6), 2000012. 10.1002/bies.202000012 32227363

[eva13223-bib-0131] Ottenburghs, J. , Honka, J. , Müskens, G. J. D. M. , & Ellegren, H. (2020). Recent introgression between Taiga Bean Goose and Tundra Bean Goose results in a largely homogeneous landscape of genetic differentiation. Heredity, 125(1–2), 73–84. 10.1038/s41437-020-0322-z 32451423 PMC7413267

[eva13223-bib-0132] Ottenburghs, J. , Kraus, R. , van Hooft, P. , van Wieren, S. , Ydenberg, R. , & Prins, H. (2017). Avian introgression in the genomic era. Avian Research, 8(1), 30.

[eva13223-bib-0133] Ottenburghs, J. , Megens, H.‐J. , Kraus, R. , Van Hooft, P. , Van Wieren, S. , Crooijmans, R. , Ydenberg, R. , Groenen, M. , & Prins, H. (2017). A history of hybrids? Genomic patterns of introgression in the True Geese. BMC Evolutionary Biology, 17(1), 201. 28830337 10.1186/s12862-017-1048-2 PMC5568201

[eva13223-bib-0134] Ottenburghs, J. , van Hooft, P. , van Wieren, S. , Ydenberg, R. , & Prins, H. (2016). Birds in a bush: Toward an avian phylogenetic network. The Auk, 133(4), 577–582.

[eva13223-bib-0135] Oziolor, E. M. , Reid, N. M. , Yair, S. , Lee, K. M. , Guberman VerPloeg, S. , Bruns, P. C. , Shaw, J. R. , Whitehead, A. , & Matson, C. W. (2019). Adaptive introgression enables evolutionary rescue from extreme environmental pollution. Science, 364(6439), 455–457. 10.1126/science.aav4155 31048485

[eva13223-bib-0136] Parmesan, C. (2006). Ecological and evolutionary responses to recent climate change. Annual Review of Ecology, Evolution, and Systematics, 37(1), 637–669. 10.1146/annurev.ecolsys.37.091305.110100

[eva13223-bib-0137] Payseur, B. (2010). Using differential introgression in hybrid zones to identify genomic regions involved in speciation. Molecular Ecology Resources, 10(5), 806–820. 10.1111/j.1755-0998.2010.02883.x 21565092

[eva13223-bib-0138] Peng, Y. , Dong, Y. , Xu, H. , Xi, X. , Niklas, K. J. , & Sun, S. (2018). Domesticated honeybees facilitate interspecific hybridization between two *Taraxacum* congeners. Journal of Ecology, 106(3), 1204–1216. 10.1111/1365-2745.12909

[eva13223-bib-0139] Peona, V. , Weissensteiner, M. , & Suh, A. (2018). How complete are ‘complete’ genome assemblies?‐An avian perspective. Molecular Ecology Resources, 18(6), 1188–1195. 30035372 10.1111/1755-0998.12933

[eva13223-bib-0140] Pfenninger, M. , Reinhardt, F. , & Streit, B. (2002). Evidence for cryptic hybridization between different evolutionary lineages of the invasive clam genus *Corbicula* (Veneroida, Bivalvia). Journal of Evolutionary Biology, 15(5), 818–829. 10.1046/j.1420-9101.2002.00440.x

[eva13223-bib-0141] Pickup, M. , Field, D. L. , Rowell, D. M. , & Young, A. G. (2013). Source population characteristics affect heterosis following genetic rescue of fragmented plant populations. Proceedings of the Royal Society B: Biological Sciences, 280(1750), 20122058. 10.1098/rspb.2012.2058 PMC3574427 23173202

[eva13223-bib-0142] Pimm, S. L. , Dollar, L. , & Bass, O. L. (2006). The genetic rescue of the Florida panther. Animal Conservation, 9(2), 115–122. 10.1111/j.1469-1795.2005.00010.x

[eva13223-bib-0143] Plomion, C. , Aury, J.‐M. , Amselem, J. , Leroy, T. , Murat, F. , Duplessis, S. , Faye, S. , Francillonne, N. , Labadie, K. , Le Provost, G. , Lesur, I. , Bartholomé, J. , Faivre‐Rampant, P. , Kohler, A. , Leplé, J.‐C. , Chantret, N. , Chen, J. , Diévart, A. , Alaeitabar, T. , … Salse, J. (2018). Oak genome reveals facets of long lifespan. Nature Plants, 4(7), 440–452. 10.1038/s41477-018-0172-3 29915331 PMC6086335

[eva13223-bib-0144] Popa‐Báez, Á. D. , Catullo, R. , Lee, S. F. , Yeap, H. L. , Mourant, R. G. , Frommer, M. , Sved, J. A. , Cameron, E. C. , Edwards, O. R. , Taylor, P. W. , & Oakeshott, J. G. (2020). Genome‐wide patterns of differentiation over space and time in the Queensland fruit fly. Scientific Reports, 10(1), 1–13. 10.1038/s41598-020-67397-5 32612249 PMC7329829

[eva13223-bib-0145] Prentis, P. J. , White, E. M. , Radford, I. J. , Lowe, A. J. , & Clarke, A. R. (2007). Can hybridization cause local extinction: A case for demographic swamping of the Australian native Senecio pinnatifolius by the invasive Senecio madagascariensis? New Phytologist, 176(4), 902–912. 10.1111/j.1469-8137.2007.02217.x 17850249

[eva13223-bib-0146] Presgraves, D. C. (2002). Patterns of postzygotic isolation in Lepidoptera. Evolution, 56(6), 1168–1183. 10.1111/j.0014-3820.2002.tb01430.x 12144018

[eva13223-bib-0147] Pritchard, J. K. , Stephens, M. , & Donnelly, P. (2000). Inference of population structure using multilocus genotype data. Genetics, 155(2), 945–959.Retrieved from http://www.ncbi.nlm.nih.gov/pubmed/10835412 10835412 10.1093/genetics/155.2.945 PMC1461096

[eva13223-bib-0148] Queirós, J. , Gortázar, C. , & Alves, P. C. (2020). Deciphering anthropogenic effects on the genetic background of the red deer in the Iberian Peninsula. Frontiers in Ecology and Evolution, 8, 147. 10.3389/fevo.2020.00147

[eva13223-bib-0149] Randi, E. (2008). Detecting hybridization between wild species and their domesticated relatives. Molecular Ecology, 17(1), 285–293. 10.1111/j.1365-294X.2007.03417.x 18173502

[eva13223-bib-0150] Ravinet, M. , Faria, R. , Butlin, R. K. , Galindo, J. , Bierne, N. , Rafajlović, M. , Noor, M. A. F. , Mehlig, B. , & Westram, A. M. (2017). Interpreting the genomic landscape of speciation: A road map for finding barriers to gene flow. Journal of Evolutionary Biology, 30(8), 1450–1477. 28786193 10.1111/jeb.13047

[eva13223-bib-0151] Reid, K. , Carlos Garza, J. , Gephard, S. R. , Caccone, A. , Post, D. M. , & Palkovacs, E. P. (2020). Restoration‐mediated secondary contact leads to introgression of alewife ecotypes separated by a colonial‐era dam. Evolutionary Applications, 13(4), 652–664. 10.1111/eva.12890 32211058 PMC7086056

[eva13223-bib-0152] Rhymer, J. M. , & Simberloff, D. (1996). Extinction by hybridization and introgression. Annual Review of Ecology and Systematics, 27(1), 83–109. 10.1146/annurev.ecolsys.27.1.83

[eva13223-bib-0153] Ruddiman, W. F. (2003). The anthropogenic greenhouse era began thousands of years ago. Climatic Change, 61(3), 261–293. 10.1023/B:CLIM.0000004577.17928.fa

[eva13223-bib-0154] Rudolfsen, T. , Ruppert, J. L. W. , Taylor, E. B. , Davis, C. S. , Watkinson, D. A. , & Poesch, M. S. (2019). Habitat use and hybridisation between the Rocky Mountain sculpin (*Cottus* sp.) and slimy sculpin (*Cottus cognatus*). Freshwater Biology, 64(3), 391–404. 10.1111/fwb.13225

[eva13223-bib-0155] Runemark, A. , Vallejo‐Marin, M. , & Meier, J. I. (2019). Eukaryote hybrid genomes. PLoS Genetics, 15(11), e1008404. 10.1371/journal.pgen.1008404 31774811 PMC6880984

[eva13223-bib-0156] Rutherford, S. , Rossetto, M. , Bragg, J. G. , McPherson, H. , Benson, D. , Bonser, S. P. , & Wilson, P. G. (2018). Speciation in the presence of gene flow: Population genomics of closely related and diverging Eucalyptus species. Heredity, 1, 10.1038/s41437-018-0073-2 PMC6039520 29632325

[eva13223-bib-0157] Saltonstall, K. , Lambert, A. M. , & Rice, N. (2016). What happens in Vegas, better stay in Vegas: Phragmites australis hybrids in the Las Vegas Wash. Biological Invasions, 18(9), 2463–2474. 10.1007/s10530-016-1167-5

[eva13223-bib-0158] Salvatori, V. , Godinho, R. , Braschi, C. , Boitani, L. , & Ciucci, P. (2019). High levels of recent wolf × dog introgressive hybridization in agricultural landscapes of central Italy. European Journal of Wildlife Research, 65(5), 1–14. 10.1007/s10344-019-1313-3

[eva13223-bib-0159] Schulte, U. , Veith, M. , & Hockkirch, A. (2012). Rapid genetic assimilation of native wall lizard populations (*Podarcis muralis*) through extensive hybridization with introduced lineages. Molecular Ecology, 21(17), 4313–4326. 10.1111/j.1365-294X.2012.05693.x 22765844

[eva13223-bib-0160] Schwenk, K. , Brede, N. , & Streit, B. (2008). Introduction. Extent, processes and evolutionary impact of interspecific hybridization in animals. Philosophical Transactions of the Royal Society of London. Series B, Biological Sciences, 363(1505), 2805–2811. 10.1098/rstb.2008.0055 18534946 PMC2453525

[eva13223-bib-0161] Scott, P. A. , Glenn, T. C. , & Rissler, L. J. (2019). Formation of a recent hybrid zone offers insight into the geographic puzzle and maintenance of species boundaries in musk turtles. Molecular Ecology, 28(4), 761–771. 10.1111/mec.14983 30578692

[eva13223-bib-0162] Seehausen, O. (2004). Hybridization and adaptive radiation. Trends in Ecology and Evolution, 19(4), 198–207. 10.1016/j.tree.2004.01.003 16701254

[eva13223-bib-0163] Seehausen, O. , Takimoto, G. , Roy, D. , & Jokela, J. (2008). Speciation reversal and biodiversity dynamics with hybridization in changing environments. Molecular Ecology, 17(1), 30–44. 10.1111/j.1365-294X.2007.03529.x 18034800

[eva13223-bib-0164] Seehausen, O. , Van Alphen, J. J. M. , & Witte, F. (1997). Cichlid fish diversity threatened by eutrophication that curbs sexual selection. Science, 277(5333), 1808–1811. 10.1126/science.277.5333.1808

[eva13223-bib-0165] Sefc, K. M. , Mattersdorfer, K. , Hermann, C. M. , & Koblmüller, S. (2017). Past lake shore dynamics explain present pattern of unidirectional introgression across a habitat barrier. Hydrobiologia, 791(1), 69–82. 10.1007/s10750-016-2791-x PMC6557712 31186578

[eva13223-bib-0166] Sendell‐Price, A. T. , Ruegg, K. C. , Anderson, E. C. , Quilodrán, C. S. , Van Doren, B. M. , Underwood, V. L. , Coulson, T. , & Clegg, S. M. (2020). The genomic landscape of divergence across the speciation continuum in island‐colonising silvereyes (*Zosterops lateralis*). G3: Genes, Genomes, Genetics, 10(9), 3147–3163. 10.1534/g3.120.401352 32660974 PMC7466963

[eva13223-bib-0167] Setter, D. , Mousset, S. , Cheng, X. , Nielsen, R. , DeGiorgio, M. , & Hermisson, J. (2020). VolcanoFinder: Genomic scans for adaptive introgression. PLoS Genetics, 16(6), e1008867. 10.1371/journal.pgen.1008867 32555579 PMC7326285

[eva13223-bib-0168] Seyoum, S. , Adams, D. H. , Matheson, R. E. , Whittington, J. A. , Alvarez, A. C. , Sheridan, N. E. , Panzner, K. , & Puchulutegui, C. (2020). Genetic relationships and hybridization among three western Atlantic sparid species: Sheepshead (*Archosargus probatocephalus*), sea bream (*A. rhomboidalis*) and pinfish (*Lagodon rhomboides*). Conservation Genetics, 21(1), 161–173. 10.1007/s10592-019-01244-7

[eva13223-bib-0169] Sivyer, L. , Morgan‐Richards, M. , Koot, E. , & Trewick, S. A. (2018). Anthropogenic cause of range shifts and gene flow between two grasshopper species revealed by environmental modelling, geometric morphometrics and population genetics. Insect Conservation and Diversity, 11(5), 415–434. 10.1111/icad.12289

[eva13223-bib-0170] Slabbekoorn, H. , Bouton, N. , van Opzeeland, I. , Coers, A. , ten Cate, C. , & Popper, A. N. (2010). A noisy spring: The impact of globally rising underwater sound levels on fish. Trends in Ecology and Evolution, 25(7), 419–427. 10.1016/j.tree.2010.04.005 20483503

[eva13223-bib-0171] Slabbekoorn, H. , & Ripmeester, E. A. P. (2008). Birdsong and anthropogenic noise: Implications and applications for conservation. Molecular Ecology, 17(1), 72–83. 10.1111/j.1365-294X.2007.03487.x 17784917

[eva13223-bib-0172] Smadja, C. , & Butlin, R. K. (2009). On the scent of speciation: The chemosensory system and its role in premating isolation. Heredity, 102(1), 77–97. 10.1038/hdy.2008.55 18685572

[eva13223-bib-0173] Song, Y. , Endepols, S. , Klemann, N. , Richter, D. , Matuschka, F.‐R. , Shih, C.‐H. , Nachman, M. W. , & Kohn, M. H. (2011). Adaptive introgression of anticoagulant rodent poison resistance by hybridization between old world mice. Current Biology, 21(15), 1296–1301. 10.1016/J.CUB.2011.06.043 21782438 PMC3152605

[eva13223-bib-0174] Steffen, W. , Broadgate, W. , Deutsch, L. , Gaffney, O. , & Ludwig, C. (2015). The trajectory of the Anthropocene: The great acceleration. The Anthropocene Review, 2(1), 81–98. 10.1177/2053019614564785

[eva13223-bib-0175] Stukenbrock, E. H. (2016). The role of hybridization in the evolution and emergence of new fungal plant pathogens. Phytopathology, 106(2), 104–112. 10.1094/PHYTO-08-15-0184-RVW 26824768

[eva13223-bib-0176] Taillebois, L. , Sabatino, S. , Manicki, A. , Daverat, F. , Nachón, D. J. , & Lepais, O. (2020). Variable outcomes of hybridization between declining *Alosa alosa* and *Alosa fallax* . Evolutionary Applications, 13(4), 636–651. 10.1111/eva.12889 32211057 PMC7086104

[eva13223-bib-0177] Taylor, S. A. , & Larson, E. L. (2019). Insights from genomes into the evolutionary importance and prevalence of hybridization in nature. Nature Ecology & Evolution, 3(2), 170–177. 10.1038/s41559-018-0777-y 30697003

[eva13223-bib-0178] Taylor, S. A. , Larson, E. L. , & Harrison, R. G. (2015). Hybrid zones: Windows on climate change. Trends in Ecology and Evolution, 30(7), 398–406. 10.1016/j.tree.2015.04.010 25982153 PMC4794265

[eva13223-bib-0179] Tibihika, P. D. , Curto, M. , Alemayehu, E. , Waidbacher, H. , Masembe, C. , Akoll, P. , & Meimberg, H. (2020). Molecular genetic diversity and differentiation of Nile tilapia (*Oreochromis niloticus*, L. 1758) in East African natural and stocked populations. BMC Evolutionary Biology, 20(1), 16. 10.1186/s12862-020-1583-0 32000675 PMC6990601

[eva13223-bib-0180] Tiesmeyer, A. , Ramos, L. , Manuel Lucas, J. , Steyer, K. , Alves, P. C. , Astaras, C. , Brix, M. , Cragnolini, M. , Domokos, C. , Hegyeli, Z. , Janssen, R. , Kitchener, A. C. , Lambinet, C. , Mestdagh, X. , Migli, D. , Monterroso, P. , Mulder, J. L. , Schockert, V. , Youlatos, D. , … Nowak, C. (2020). Range‐wide patterns of human‐mediated hybridisation in European wildcats. Conservation Genetics, 21(2), 247–260. 10.1007/s10592-019-01247-4

[eva13223-bib-0181] Todesco, M. , Pascual, M. A. , Owens, G. L. , Ostevik, K. L. , Moyers, B. T. , Hübner, S. , Heredia, S. M. , Hahn, M. A. , Caseys, C. , Bock, D. G. , & Rieseberg, L. H. (2016). Hybridization and extinction. Evolutionary Applications, 9(7), 892–908. 10.1111/eva.12367 27468307 PMC4947151

[eva13223-bib-0182] Valencia‐Montoya, W. A. , Elfekih, S. , North, H. L. , Meier, J. I. , Warren, I. A. , Tay, W. T. , Gordon, K. H. J. , Specht, A. , Paula‐Moraes, S. V. , Rane, R. , Walsh, T. K. , & Jiggins, C. D. (2020). Adaptive introgression across semipermeable species boundaries between local Helicoverpa zea and invasive Helicoverpa armigera moths. Molecular Biology and Evolution, 37(9), 2568–2583. 10.1093/molbev/msaa108 32348505 PMC7475041

[eva13223-bib-0183] Vallejo‐Marín, M. , & Hiscock, S. J. (2016). Hybridization and hybrid speciation under global change. The New Phytologist, 211(4), 1170–1187. 10.1111/nph.14004 27214560

[eva13223-bib-0184] van Hengstum, T. , Lachmuth, S. , Oostermeijer, J. G. B. , den Nijs, J. C. M. , Meirmans, P. G. , & van Tienderen, P. H. (2012). Human‐induced hybridization among congeneric endemic plants on Tenerife, Canary Islands. Plant Systematics and Evolution, 298(6), 1119–1131. 10.1007/s00606-012-0624-6

[eva13223-bib-0185] van Wyk, A. M. , Dalton, D. L. , Hoban, S. , Bruford, M. W. , Russo, I.‐R.‐M. , Birss, C. , Grobler, P. , van Vuuren, B. J. , & Kotzé, A. (2017). Quantitative evaluation of hybridization and the impact on biodiversity conservation. Ecology and Evolution, 7(1), 320–330. 10.1002/ece3.2595 28070295 PMC5214875

[eva13223-bib-0186] van Wyk, A. M. , Dalton, D. L. , Kotzé, A. , Grobler, J. P. , Mokgokong, P. S. , Kropff, A. S. , & Jansen van Vuuren, B. (2019). Assessing introgressive hybridization in roan antelope (*Hippotragus equinus*): Lessons from South Africa. PLoS One, 14(10), e0213961. 10.1371/journal.pone.0213961 31626669 PMC6799913

[eva13223-bib-0187] Vaux, F. , Bohn, S. , Hyde, J. R. , & O’Malley, K. G. (2021). Adaptive markers distinguish North and South Pacific Albacore amid low population differentiation. Evolutionary Applications, eva.13202. 10.1111/eva.13202 PMC8127716 34025772

[eva13223-bib-0188] vonHoldt, B. M. , Brzeski, K. E. , Wilcove, D. S. , & Rutledge, L. Y. (2018). Redefining the Role of Admixture and Genomics in Species Conservation. Conservation Letters, 11(2), e12371. 10.1111/conl.12371

[eva13223-bib-0189] Vonlanthen, P. , Bittner, D. , Hudson, A. G. , Young, K. A. , Müller, R. , Lundsgaard‐Hansen, B. , Roy, D. , Di Piazza, S. , Largiader, C. R. , & Seehausen, O. (2012). Eutrophication causes speciation reversal in whitefish adaptive radiations. Nature, 482(7385), 357–362. 10.1038/nature10824 22337055

[eva13223-bib-0190] Waters, C. N. , Zalasiewicz, J. , Summerhayes, C. , Barnosky, A. D. , Poirier, C. , Ga uszka, A. , Cearreta, A. , Edgeworth, M. , Ellis, E. C. , Ellis, M. , Jeandel, C. , Leinfelder, R. , McNeill, J. R. , Richter, D. D. , Steffen, W. , Syvitski, J. , Vidas, D. , Wagreich, M. , Williams, M. , … Wolfe, A. P. (2016). The Anthropocene is functionally and stratigraphically distinct from the Holocene. Science, 351(6269), aad2622. 10.1126/science.aad2622 26744408

[eva13223-bib-0191] Weeks, A. R. , Heinze, D. , Perrin, L. , Stoklosa, J. , Hoffmann, A. A. , Van Rooyen, A. , Kelly, T. , & Mansergh, I. (2017). Genetic rescue increases fitness and aids rapid recovery of an endangered marsupial population. Nature Communications, 8(1), 1–6. 10.1038/s41467-017-01182-3 PMC5715156 29057865

[eva13223-bib-0192] Whiteley, A. R. , Fitzpatrick, S. W. , Funk, W. C. , & Tallmon, D. A. (2015). Genetic rescue to the rescue. Trends in Ecology and Evolution, 30(1), 42–49. 10.1016/j.tree.2014.10.009 25435267

[eva13223-bib-0193] Whitney, K. D. , Randell, R. A. , & Rieseberg, L. H. (2006). Adaptive introgression of herbivore resistance traits in the weedy sunflower *Helianthus annuus* . The American Naturalist, 167(6), 794–807. 10.1086/504606 16649157

[eva13223-bib-0194] Wielstra, B. , Burke, T. , Butlin, R. K. , Schaap, O. , Shaffer, H. B. , Vrieling, K. , & Arntzen, J. W. (2016). Efficient screening for ‘genetic pollution’ in an anthropogenic crested newt hybrid zone. Conservation Genetics Resources, 8(4), 553–560. 10.1007/s12686-016-0582-3

[eva13223-bib-0195] Willoughby, J. R. , Fernandez, N. B. , Lamb, M. C. , Ivy, J. A. , Lacy, R. C. , & DeWoody, J. A. (2015). The impacts of inbreeding, drift and selection on genetic diversity in captive breeding populations. Molecular Ecology, 24(1), 98–110. 10.1111/mec.13020 25443807

[eva13223-bib-0196] Wolf, D. E. , Takebayashi, N. , & Rieseberg, L. H. (2001). Predicting the risk of extinction through hybridization. Conservation Biology, 15(4), 1039–1053. 10.1046/j.1523-1739.2001.0150041039.x

[eva13223-bib-0197] Wolf, J. B. W. , & Ellegren, H. (2017). Making sense of genomic islands of differentiation in light of speciation. Nature Reviews Genetics, 18(2), 87–100. 10.1038/nrg.2016.133 27840429

[eva13223-bib-0198] Wooten, J. A. , Sullivan, B. K. , Klooster, M. R. , Schwaner, T. D. , Sullivan, K. O. , Brown, A. D. , Takahashi, M. , & Bradford, P. R. (2019). Thirty years of hybridization between toads along the Agua Fria River in Arizona: Part II: Fine‐scale assessment of genetic changes over time using microsatellites. Journal of Herpetology, 53(2), 104. 10.1670/18-101

[eva13223-bib-0199] Wu, C.‐I. (2001). The genic view of the process of speciation. Journal of Evolutionary Biology, 14(6), 851–865.

[eva13223-bib-0200] Zhang, L. , Thibert‐Plante, X. , Ripa, J. , Svanbäck, R. , & Brännström, Å. (2019). Biodiversity loss through speciation collapse: Mechanisms, warning signals, and possible rescue. Evolution, 73(8), 1504–1516. 10.1111/evo.13736 30980527

[eva13223-bib-0201] Zhang, X. , Kim, B. , Lohmueller, K. E. , & Huerta‐Sánchez, E. (2020). The impact of recessive deleterious variation on signals of adaptive introgression in human populations. Genetics, 215(3), 799–812. 10.1534/genetics.120.303081 32487519 PMC7337073

[eva13223-bib-0202] Zhou, X. , Lutteropp, S. , Czech, L. , Stamatakis, A. , Looz, M. V. , & Rokas, A. (2020). Quartet‐based computations of internode certainty provide robust measures of phylogenetic incongruence. Systematic Biology, 69(2), 308–324. 10.1093/sysbio/syz058 31504977

